# Multi-Omics Analysis Reveals Hormonal, miRNA-Associated, and NLR-Linked Aphid Defense in *Lycium barbarum*

**DOI:** 10.3390/plants15203097

**Published:** 2026-10-10

**Authors:** Ting Yan, Zi-Kai Zhou, Peng-Fei Shi, Jing-Yu Yue, Hao Sang, Ru-Ru Shi, Shu-Juan Chang, Long Hai

**Affiliations:** 1 Key Laboratory of National Forestry and Grassland Administration on Sandy Biological Resources Conservation, Cultivation and Utilization, Inner Mongolia Academy of Forestry Sciences, Hohhot 010010, China; nmgyanting@163.com (T.Y.);; 2 Crop Research Institute, Anhui Academy of Agricultural Sciences, Hefei 230031, China; 67983286@163.com

**Keywords:** *Lycium barbarum*, aphid resistance, multi-omics, salicylic acid, jasmonic acid, microRNA, *NLR* genes, WGCNA

## Abstract

Aphid infestation constrains goji berry production, yet its defense architecture remains poorly resolved. We integrated comparative transcriptomics, phytohormone profiling, small RNA sequencing, and genome-wide nucleotide-binding leucine-rich repeat (*NLR*) analysis to dissect aphid defense in *Lycium barbarum* (cv. Ningqi-10)and susceptible *L. chinense*. Aphid bioassays confirmed that *L. barbarum* imposes a significant fitness cost on aphids, reducing survival, longevity, and fecundity. Transcriptomic comparison revealed fewer but more defense-enriched differentially expressed genes in the resistant accession. Weighted gene co-expression network analysis (WGCNA) identified a stress-associated turquoise module (288 genes, r = 0.981) receptor-like kinases and mitogen-activated protein kinase (MAPK) components, bridging extracellular signals sensing and hormone signaling. Phytohormone profiling showed that salicylic acid (SA) accumulated to 72 h, whereas jasmonic acid (JA) peaked transiently at 24 h, producing an increased SA/JA ratio suggesting a shift to SA-dominated signaling. *MiR398a-3p* and *miR319-3p* dynamics coincided with this SA/JA shift, suggesting a possible link to hormone-coordinated regulation and candidate targets for validation. *NLR* analysis identified 151 candidate genes, including 18 *L. barbarum*-biased *NLRs* up-regulation under SA-dominated signaling, implying a link to SA signaling. Collectively, these findings support a multi-layered framework integrating SA signaling, transcriptional reprogramming, and coupled miRNA and *NLR* responses in aphid defense.

## 1. Introduction

The genus *Lycium* (*Solanaceae*) comprises approximately 80 species distributed across temperate and subtropical regions, many of which are valued for their nutritional and medicinal properties [1]. Among these, *Lycium barbarum* L. (goji berry or wolfberry) is an economically important medicinal and edible tree species in China, with commercial plantations in Northwest China supplying over 90% of the global production. Driven by sustained international demand for functional foods rich in polysaccharides, carotenoids, and flavonoids, the annual output value of this industry has reached billions of yuan [2,3]. Ningxia alone cultivates more than 67,000 hectares of *L. barbarum*, making goji berry a cornerstone of the region’s agricultural economy [4].

Despite its economic significance, goji berry (*Lycium* spp.) production faces a persistent threat from aphid infestation. Among the aphid species attacking goji, the goji-specialized strain of *Aphis gossypii* Glover is the dominant pest, accounting for 87% of *Aphis* sp., followed by the green peach aphid (*Myzus persicae* Sulzer) and the cowpea aphid (*Aphis craccivora* Koch) [5,6]. These aphids colonize young leaves, shoot tips, and developing fruits, causing damage through direct phloem sap feeding and indirect transmission of plant viruses [7,8]. Field surveys conducted in the major goji-producing regions of Ningxia and Qinghai have documented yield losses ranging from 20% to 40% during peak infestation periods [4]. Current control practices rely heavily on the application of synthetic insecticides; however, repeated use has driven the evolution of insecticide-resistant aphid populations [9], disrupted natural enemy communities, as also documented in other crop systems [10] and raised public concern over chemical residues in edible berries [11,12]. Collectively, these challenges underscore the urgent need to develop sustainable management strategies informed by a thorough molecular understanding of natural defense mechanisms in goji.

Plant defense against aphids is orchestrated at multiple regulatory layers. Phytohormones, particularly salicylic acid (SA) and jasmonic acid (JA), act as central signaling hubs, with SA frequently dominating responses to phloem-feeding insects and JA driving wound- and herbivore-induced defense programs [9,13,14,15,16,17]. In addition to its role in plant immunity, SA has been shown to directly suppress the immune responses of phytophagous insects by interrupting the universal immune mediator dorsal switch protein 1 (DSP1) [18], highlighting a tritrophic defensive role for this hormone. Their interaction is highly context-dependent, ranging from synergistic to mutually suppressive depending on the herbivore, tissue, and temporal dynamics [19,20,21,22,23,24]. At the post-transcriptional level, microRNAs (miRNAs) add an additional layer of regulation, with specific miRNAs such as *miR393*, *miR319*, *miR398*, and *miR156* modulating hormone signaling and defense gene expression [25,26,27,28,29,30]. Importantly, these regulatory layers are interconnected: hormone signals influence miRNA expression, miRNAs in turn modulate hormone pathway components and both converge on transcriptional reprogramming that shapes resistance outcomes [24,31]. In addition, hormone signaling may also be linked to the responses of nucleotide-binding leucine-rich repeat (*NLR*) immune receptors, as the expression or activation of these intracellular receptors may be modulated by upstream hormonal signals.

Despite broad conservation of these SA/JA and miRNA modules across angiosperms, their deployment in woody perennials such as *Lycium* remains poorly understood. Unlike annual models, *Lycium* is a perennial shrub that must balance defense with multi-year growth and fruit production, potentially imposing distinct selective pressures on defense signaling. Moreover, *Lycium* species accumulate abundant phenolic compounds and polysaccharides that may intersect with SA- and JA-related pathways, raising the possibility of lineage-specific regulatory configurations [32]. The availability of two closely related *Lycium* accessions with contrasting aphid resistance—the resistant *L. barbarum* (cv. Ningqi-10) and the susceptible *L. chinense*—provides a natural comparative system to dissect how conserved defense modules are deployed within this economically important genus.

Against this backdrop, however, molecular research on aphid defense in *Lycium* remains in its infancy, despite the availability of genome assemblies for *L. barbarum* and *L. ruthenicum* [2,3,33,34]. Functional studies are largely limited to phenotypic observations and broad physiological measurements. By contrast, in major crops and model systems, extensive transcriptomic and genomic studies have established that aphid feeding reshapes defense signaling networks, stress-responsive transcription, and secondary metabolism [35,36,37,38,39,40]. Comparative studies in chrysanthemum and tomato have further revealed that aphid resistance involves broad transcriptional remodeling, proteomic changes, and even grafting-associated effects [41,42,43]. In *Lycium*, recent work has reported JA and SA crosstalk in interactions between *A. gossypii* and *L. barbarum* [32]. However, an integrated view encompassing transcriptomic reprogramming, phytohormone dynamics, miRNA-associated post-transcriptional regulation and *NLR* immune receptor responses is still lacking. Despite these advances, how SA/JA signaling, miRNA-associated regulation, and *NLR* immune receptor responses are temporally coordinated during aphid defense—particularly in woody perennials with contrasting resistance phenotypes—remains poorly understood. In this study, we integrated comparative transcriptomics, small RNA sequencing, targeted phytohormone profiling, and genome-wide *NLR* analysis to characterize aphid defense mechanisms in *L. barbarum* (cv. Ningqi-10), which exhibits stronger defense relative to the susceptible *L. chinense*. By resolving the temporal dynamics of hormonal and post-transcriptional regulation in the resistant accession and identifying accession-biased *NLR* candidates, this study provides a molecular framework for understanding aphid defense in goji and prioritizes candidate targets for resistance breeding. To this end, we performed a 72-h comparative transcriptome analysis of the two accessions, combined with WGCNA and *NLR* annotation. In parallel, to capture early transient regulatory events, we performed hormone and small RNA time-course analyses in the resistant accession *L. barbarum*; since hormone and miRNA time-course analyses were performed only in the resistant accession *L. barbarum*, the hormone and miRNA dynamics described here reflect the response of that accession alone. We therefore used an integrated approach—comparative transcriptomics, WGCNA, phytohormone profiling, small RNA sequencing, and genome-wide *NLR* analysis—to sequentially resolve transcriptomic divergence between the two accessions under aphid stress; identify accession-biased transcriptional patterns, stress-associated co-expression modules and hub genes, and candidate *NLR* responses; characterize the *NLR* gene repertoire and its expression response; and dissect the temporal dynamics of phytohormone signaling and miRNA-associated regulation in the resistant accession. By integrating these data layers, we constructed a working model linking co-expression modules, hormone dynamics, miRNA regulation, and *NLR*-associated responses, thereby providing a molecular framework for future functional validation and marker-assisted breeding in goji berry and related Solanaceae crops.

## 2. Results

### 2.1. Aphid Infection Induces Contrasting Transcriptional Programs in Resistant and Susceptible Lycium

**Figure 1:**
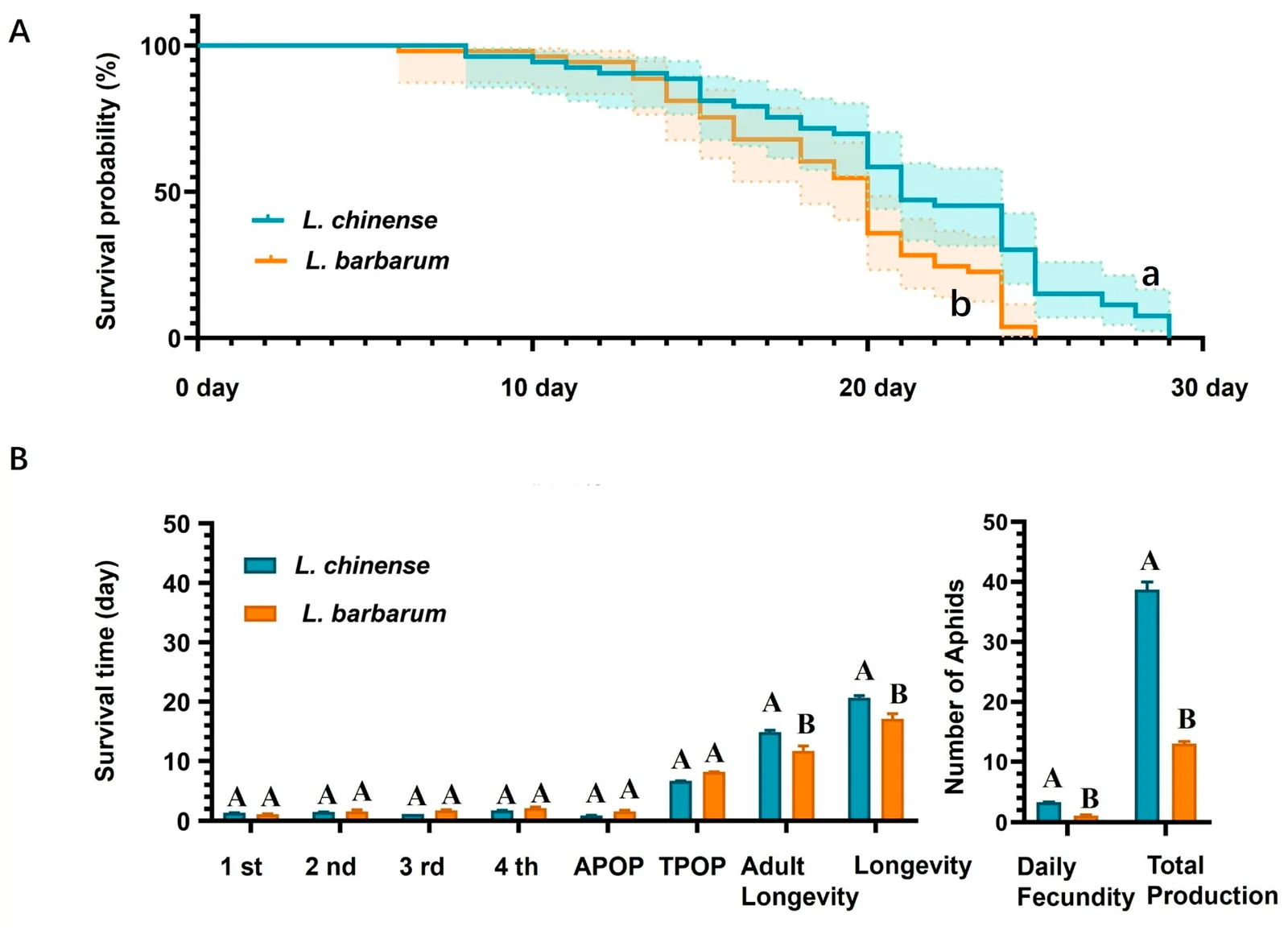
Kaplan–Meier survival curves and life table parameters of *Aphis gossypii* on *L. barbarum* and *L. chinense*. (**A**) First-instar nymphs were separately transferred onto leaves of the two *Lycium* accessions. Kaplan–Meier survival curves were generated, and pairwise curve comparisons were performed using the log-rank test. Different lowercase letters (*L. chinense* (a), *L. barbarum* (b)) indicate significant differences between curves (*p* < 0.05). Each treatment group consisted of three biological replicates, with 25 aphids per replicate. (**B**) Life table parameters of *A. gossypii* on the two hosts, including stage-specific longevity, adult longevity, total longevity, adult preoviposition period (APOP), total preoviposition period (TPOP), as well as daily fecundity and total progeny production. Data are presented as mean ± SEM (*n* = 3 biological replicates, 25 aphids per replicate). Comparisons among treatments were performed using one-way ANOVA followed by Tukey’s HSD post-hoc test; different uppercase letters (A, B) indicate significant differences (*p* < 0.05).

To quantify the resistance difference between the two accessions, we first constructed age-stage, two-sex life tables for-*A. gossypii*-feeding on-*L. barbarum*-(cv. Ningqi-10) and *L. chinense*. The survival curves of aphids on the two hosts differed significantly (log-rank test, *p* < 0.05), with aphids fed on the resistant *L. barbarum* exhibiting significantly shortened longevity and reduced fecundity (Figure 1A,B). Life table analysis further revealed that the net reproductive rate (*R*_0_), intrinsic rate of increase (*r_m_*), and mean generation time (*T*) were all significantly lower on *L. barbarum* than on *L. chinense* (Table S1). The adult preoviposition period (APOP) and total preoviposition period (TPOP) were also recorded and are presented in Figure 1B. These results demonstrate that *L. barbarum* (cv. Ningqi-10) imposes a substantial fitness cost on aphids, thereby suppressing their population growth potential.

**Figure 2:**
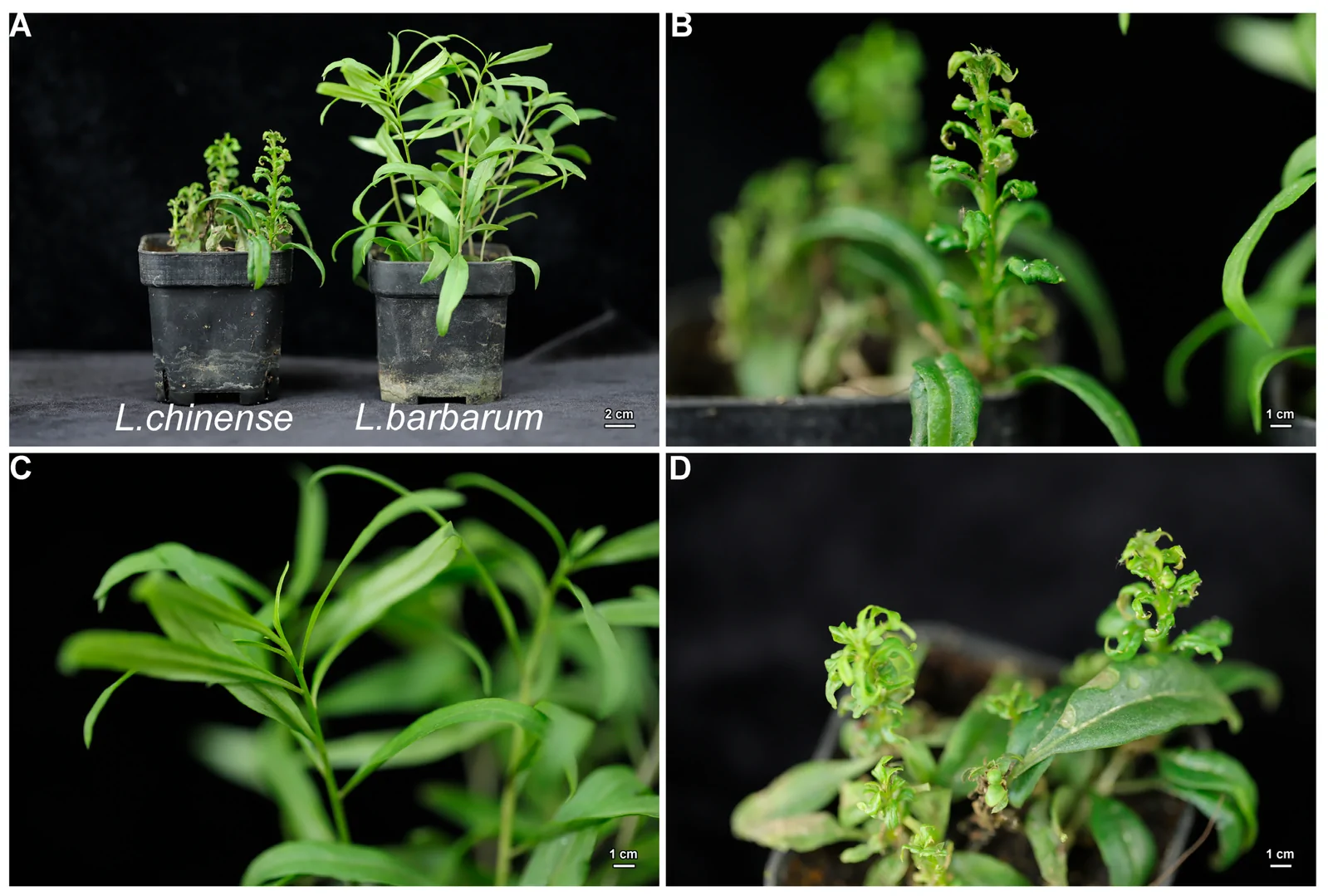
Divergent phenotypic responses of *L. barbarum* and *L. chinense* to aphid infestation. (**A**) Whole-plant phenotypes of aphid-infested *L. chinense* (**left**) and *L. barbarum* (cv. Ningqi-10; (**right**)), showing more pronounced chlorosis and leaf and shoot deformation in *L. chinense*. (**B**) Close-up of the deformed and curled shoot apex of *L. chinense*. (**C**) Close-up of the comparatively mild *L. barbarum* phenotype, with limited leaf curling. (**D**) Dense aphid colonization associated with severe leaf and shoot deformation in *L. chinense*. Scale bars, 2 cm (**A**) and 1 cm (**B**–**D**).

Consistent with these life-table parameters, clear phenotypic differences were observed at 40 days post-infestation: *L. barbarum* (cv. Ningqi-10) showed only mild chlorosis and limited leaf curling, whereas *L. chinense* exhibited pronounced leaf deformation, extensive chlorosis, and dense aphid colonization (Figure 2A–D). These contrasting later phenotypes motivated analysis of the early transcriptional programs underlying the divergence.

**Figure 3:**
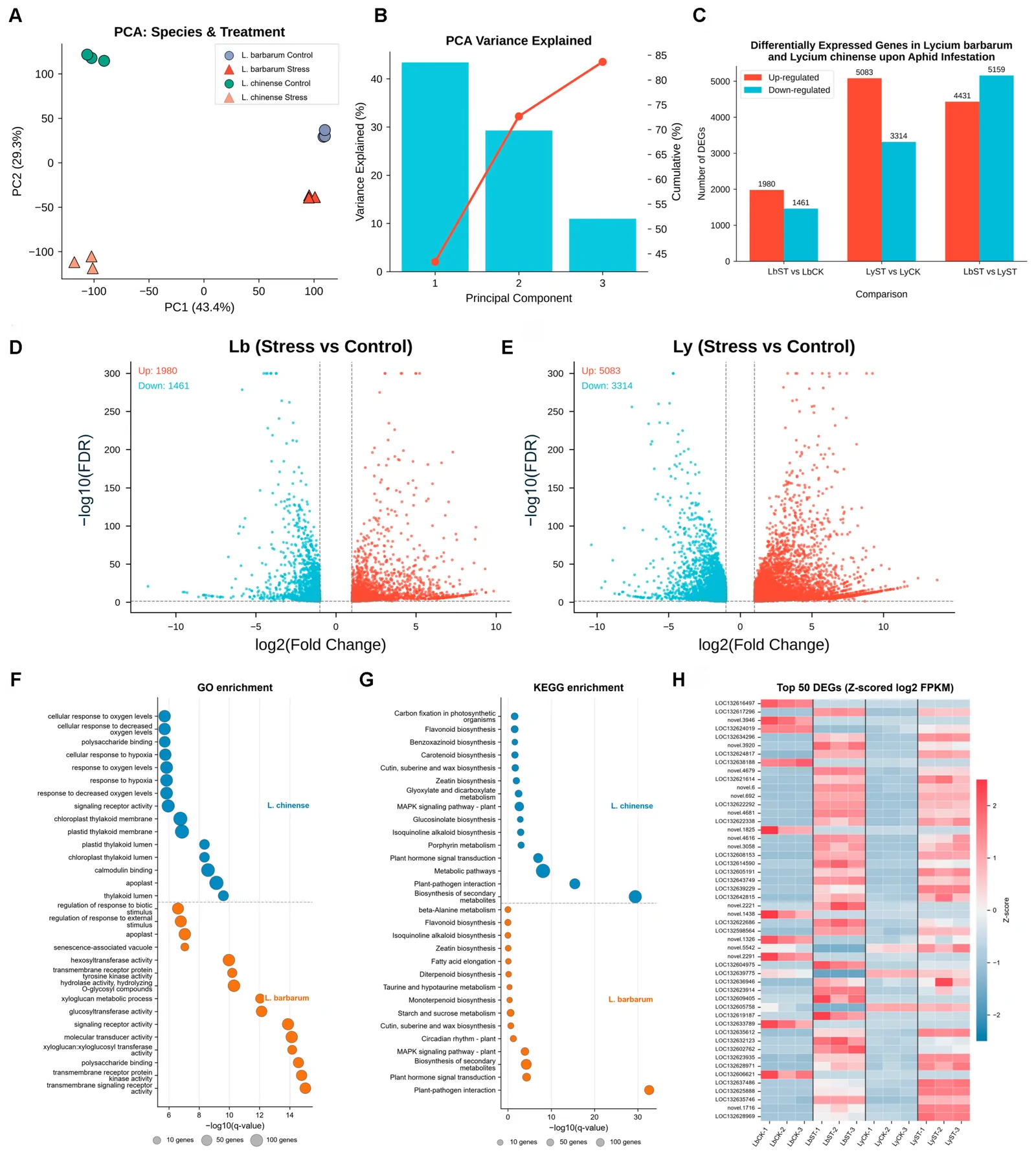
Transcriptomic responses of *L. barbarum* and *L. chinense* to aphid infestation. (**A**) Principal component analysis of 12 RNA-seq libraries. (**B**) Variance explained by PC1–PC3 and cumulative variance. (**C**) Numbers of up- and down-regulated differentially expressed genes (DEGs) in the LbST versus LbCK, LyST versus LyCK, and LbST versus LyST comparisons. (**D**) Volcano plots for the within-accession stress comparisons in *L. barbarum*. (**E**) Volcano plots for the within-accession stress comparisons in *L. chinense*. (**F**) GO and KEGG enrichment results for LbST versus LbCK. (**G**) GO and KEGG enrichment results for LyST versus LyCK. (**H**) Heatmap of the top 50 regulated DEGs.

At 72 h post-infestation, no visible phenotypic differences were observed between aphid-infested and control plants of either accession, consistent with the use of this early time point to capture transcriptional and signaling events preceding symptom development. We therefore performed comparative transcriptome analysis at 72 h. RNA-seq of both species yielded 12 libraries with an average of 42.6 million clean reads per sample (Q30 > 92%, mapping rate > 85%). Principal component analysis separated samples by genotype and treatment; PC1, PC2, and PC3 explained 43.4%, 29.3%, and 11.1% of the variance, respectively, accounting for 83.8% cumulatively (Figure 3A,B). DESeq2 analysis identified 3441 DEGs (1980 up-regulated and 1461 down-regulated) in *L. barbarum* and 8397 DEGs (5083 up-regulated and 3314 down-regulated) in *L. chinense* under aphid stress (Figure 3C–E and Figure S1A). Comparison of the DEG sets between the two accessions identified 1901 shared DEGs, together with 1540 genes detected only in the *L. barbarum* contrast and 6496 genes detected only in the *L. chinense* contrast (Figure S1B). These counts summarize the results of separate within-accession differential-expression tests and were therefore treated as descriptive accession-specific DEG patterns rather than as formal evidence that the response magnitudes differed between accessions. To directly test genotype-dependent responses, we fitted a factorial DESeq2 model including genotype, treatment, and their interaction. The interaction coefficient represented the difference in aphid-induced response between accessions, calculated as (LyST − LyCK) − (LbST − LbCK). This analysis identified 13,097 genes with a significant interaction effect at FDR < 0.05, including 8419 genes that also showed an absolute interaction log_2_ fold change ≥ 1. Thus, genotype-dependent response differences were inferred from the formal interaction test rather than from within-accession significance alone. In addition, direct cross-accession comparison of expression levels under stress (LbST versus LyST) identified 4431 genes with higher expression and 5159 with lower expression in *L. barbarum* (Figure 3C). The log_2_ fold-change distributions for the accession-specific contrasts are summarized in Figure S1C and the complete differential-expression results are provided in Table S2.

Functional enrichment analysis (Figure 3F,G; Table S3) revealed that in *L. barbarum*, phenylpropanoid metabolic and biosynthetic processes were significantly enriched (GO:0009698, 61 genes, FDR = 2.06 × 10^−4^; GO:0009699, 54 genes, FDR = 2.81 × 10^−4^), and plant-pathogen interaction was the strongest KEGG term (ko04626, 245 genes, FDR = 2.31 × 10^−33^). The *L. chinense* response also showed broad enrichment of defense-associated categories, including phenylpropanoid metabolic process (GO:0009698, 120 genes, FDR = 4.31 × 10^−5^) and plant-pathogen interaction (ko04626, 393 genes, FDR = 3.49 × 10^−16^), indicating that the susceptible genotype mounted a broader transcriptional response rather than lacking defense-term enrichment. A heatmap of the top 50 most strongly regulated DEGs showed distinct patterns, with defense genes *PR1* (*LOC132605191*, FC = 8.38), *WRKY70* (*LOC132605276*, FC = 5.37), and *CHS* (*LOC132640283*, FC = −1.30) exhibiting accession-biased responses (Figure 3H). Mean-FPKM profiles at 72 h likewise showed increased *PR4*, *LOX*, *GST*, *WRKY70*, and *PTI5* expression, together with decreased *CHS* and *PAL* expression, in the aphid-stressed resistant accession (Figure S2).

### 2.2. WGCNA Associates a Turquoise Co-Expression-Module with Stress Treatment and Defense Responses

**Figure 4:**
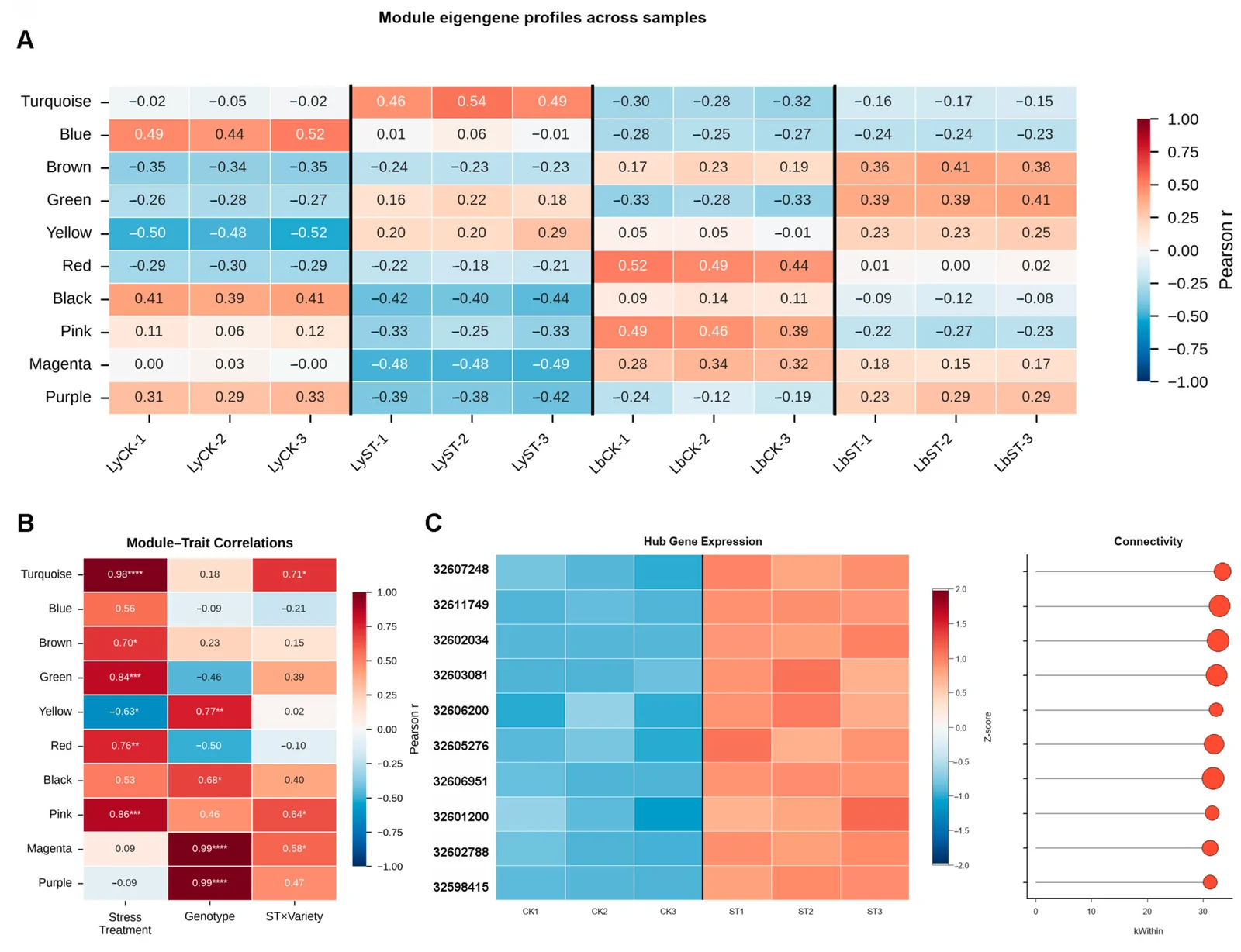
WGCNA summary of aphid-responsive co-expression patterns. (**A**) Module eigengene profiles across 12 RNA-seq samples, illustrating sample-level variation among modules. (**B**) Associations of module eigengenes with stress treatment, genotype, and their interaction. Cell values are Pearson correlation coefficients (*r*), and asterisks indicate nominal two-sided significance (* *p* < 0.05, ** *p* < 0.01, *** *p* < 0.001, and **** *p* < 0.0001). (**C**) Expression profiles and intramodular connectivity of selected turquoise-module hub genes. Together, the panels identify stress-associated modules and highly connected candidate genes.

Having established the transcriptional divergence between the two accessions, we performed WGCNA to link transcriptomic profiles to treatment and genotype. Following Langfelder and Horvath [44], genes with a coefficient of variation > 0.1 were retained, and a signed hybrid network was constructed using a soft-thresholding power of 6 (scale-free R^2^ = 0.933; Figure S3A), which led to the identification of 10 co-expression modules were identified: turquoise (288 genes), blue (94), brown (276), green (78), yellow (55), red (2073), black (756), pink (66), magenta (3991), and purple (323) (Figure S3B; Table S3). The module eigengene profiles displayed distinct stress- and accession-associated patterns across the 12 RNA-seq samples (Figure 4A).

Module eigengenes were correlated with stress treatment (ST), genotype, and their interaction using Pearson correlation. The turquoise module showed the strongest association with stress treatment (r = 0.981, *p* < 0.0001), followed by pink (r = 0.86), green (r = 0.84), red (r = 0.76), and brown (r = 0.70), indicating stress-responsive expression patterns (Figure 4B). The magenta and purple modules correlated strongly with genotype (r = 0.994 and r = 0.991, respectively), capturing constitutive differences between *L. barbarum* and *L. chinense*. The yellow module showed dual associations, negatively with stress (r = −0.63) and positively with genotype (r = 0.77), whereas the black module correlated moderately with both variables (Figure 4B; Table S3).

Among the turquoise-module hub genes, GO over-representation analysis identified defense response (25 genes, FDR = 6.21 × 10^−7^) and response to stress (29 genes, FDR = 3.16 × 10^−3^) as significantly over-represented categories, and KEGG analysis identified plant–pathogen interaction as significantly over-represented (13 genes, FDR = 8.52 × 10^−7^). Biosynthesis of secondary metabolites (9 genes) and plant MAPK signaling (3 genes) were also represented among the hub genes, although these categories did not reach significance after FDR correction. These findings indicate pathway-level associations of the hub-gene set, but do not establish causal functions.

To further dissect the functional relevance of this stress-associated module, we identified hub genes within the turquoise module based on intramodular connectivity (kWithin). The ten selected turquoise-module hub genes ranked by intramodular connectivity (kWithin) are listed in Table S6. *LOC132607248* (kWithin = 33.48) is annotated as a *CRK25*-like receptor-like kinase candidate, whereas *LOC132611749* is associated with diterpenoid biosynthesis; *LOC132602034*, *LOC132603081*, and *LOC132606200* are additional highly connected examples. The presence of receptor-like kinase and defense-associated candidates among these hub genes is consistent with the involvement of the turquoise module in defense signaling (Figure 4C). To validate the transcriptomic findings, RT-qPCR analysis of five selected genes—including *PR4*, *LOX*, *GST*, *WRKY70*, and *PTI5*—showed expression trends consistent with the RNA-seq results (Supplementary Figure S4 and Table S6). All aphid-stress responses were significant (Holm-adjusted *p* < 0.05).

### 2.3. NLR Repertoire Shows Accession-Biased Transcriptional Activation

**Figure 5:**
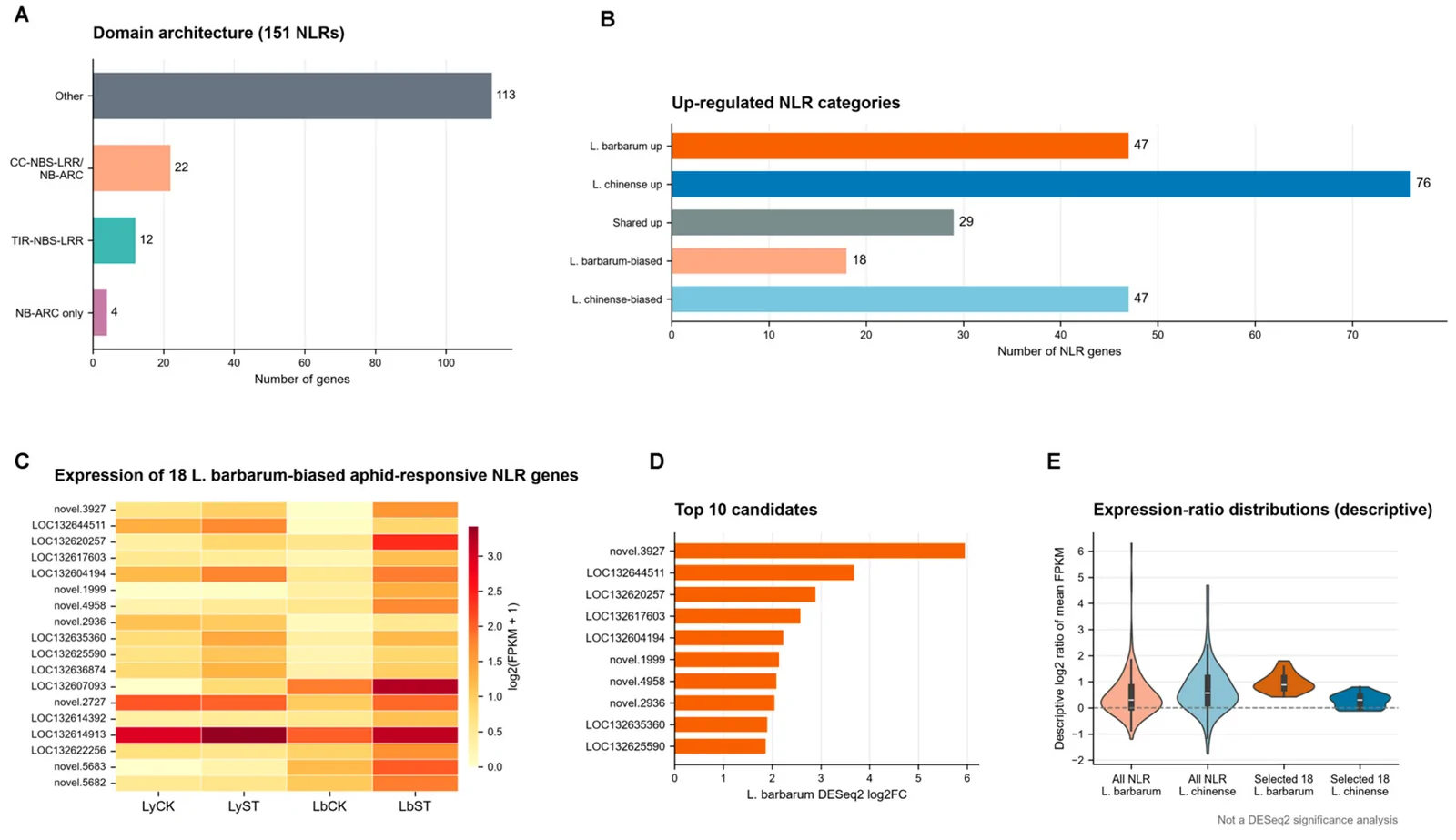
NLR-associated transcriptional responses in *L. barbarum* and *L. chinense*. (**A**) Domain-architecture distribution of the 151 candidate NLRs identified in the *L. barbarum* genome. (**B**) Overview of upregulated *NLR* categories, showing shared and accession-biased expression subsets between the two species. Here, *NLR* candidates labeled as *L. barbarum*-biased merely represent aphid-responsive genes showing expression bias in *L. barbarum* under the current cross-species mapping strategy, and should not be regarded as species-specific *NLR* genes. (**C**) Heatmap displaying the expression profiles (log_2_ mean FPKM + 1) of the 18 *L. barbarum*-biased aphid-responsive *NLR* candidates across the four treatment groups (LyCK, LyST, LbCK, LbST; *n* = 3 biological replicates per group). (**D**) Top 10 candidates ranked by DESeq2 log_2_ fold change in *L. barbarum*. (**E**) Distributions of the descriptive expression ratio, calculated as log_2_[(mean stress FPKM + 1)/(mean control FPKM + 1)], for all 151 candidate *NLRs* and for the selected 18-gene set in each accession. Note that the ratios in (**E**) are descriptive and do not represent significance-tested DESeq2 results; significance thresholds are shown in (**A**,**D**).

NLR proteins are important components of plant innate immunity, functioning as intracellular receptors that detect pathogen effectors and activate defense responses [45,46]. Given that NLR-type immune receptors may exhibit differential expression patterns between accessions, we investigated whether they show accession-biased responses in addition to the transcriptional modules described above. To this end, we performed genome-wide NLR identification and expression analysis using conserved domain information and aphid-responsive transcriptome data. Based on NB-ARC-containing sequences and domain architecture classification, a total of 151 candidate *NLR* genes were identified, with the major domain categories being other domain architectures (113 genes), *CC-NBS-LRR/NB-ARC* (22 genes), *TIR-NBS-LRR* (12 genes), and *NB-ARC*-only (4 genes) (Figure 5A; Table S7).

Comparative analysis of up-regulated *NLRs* following aphid infestation showed that 47 *NLRs* were significantly up-regulated in *L. barbarum* (log_2_FC > 1, padj < 0.05), whereas 76 met the same criterion in *L. chinense*. 29 *NLRs* were shared between the two accessions and 18 were *L. barbarum*-biased and 47 were *L. chinense*-biased (Figure 5B). Notably, these 18 *L. barbarum*-biased candidates were significantly up-regulated in *L. barbarum* but did not reach the same significance threshold in *L. chinense.* They are therefore described as *L. barbarum*-biased aphid-responsive *NLR* candidates, reflecting only accession-biased expression under the cross-species mapping strategy and should not be regarded as species-specific or genome-specific genes (Figure 5B; Table S8). The corresponding log_2_FC and adjusted *p*-values for *L. chinense* are provided in Table S8 to allow independent evaluation of the comparative expression patterns. The expression profiles of these 18 candidates are shown in Figure 5C and the top 10 candidates ranked by fold change are presented in Figure 5D. Functional annotation of the 18 *L. barbarum*-biased candidates identified NB-ARC-containing proteins with TIR-NBS-LRR and CC-NBS-LRR/NB-ARC domain architectures (Table S8), which were interpreted cautiously against characterized plant NLR immune modules [47,48,49]. Figure 5E presents the stress/control ratios based on mean FPKM for all 151 candidate *NLRs* and the 18-gene set in each accession, providing a visual comparison of expression trends; statistical significance is assessed in Figure 5A,D. Collectively, these results define an accession-biased *NLR*-associated transcriptional pattern after aphid infestation and prioritize the 18 *L. barbarum*-biased aphid-responsive *NLR* candidates for functional validation of aphid resistance.

### 2.4. Sustained SA Accumulation and Transient JA Burst Define Hormonal Dynamics

**Figure 6:**
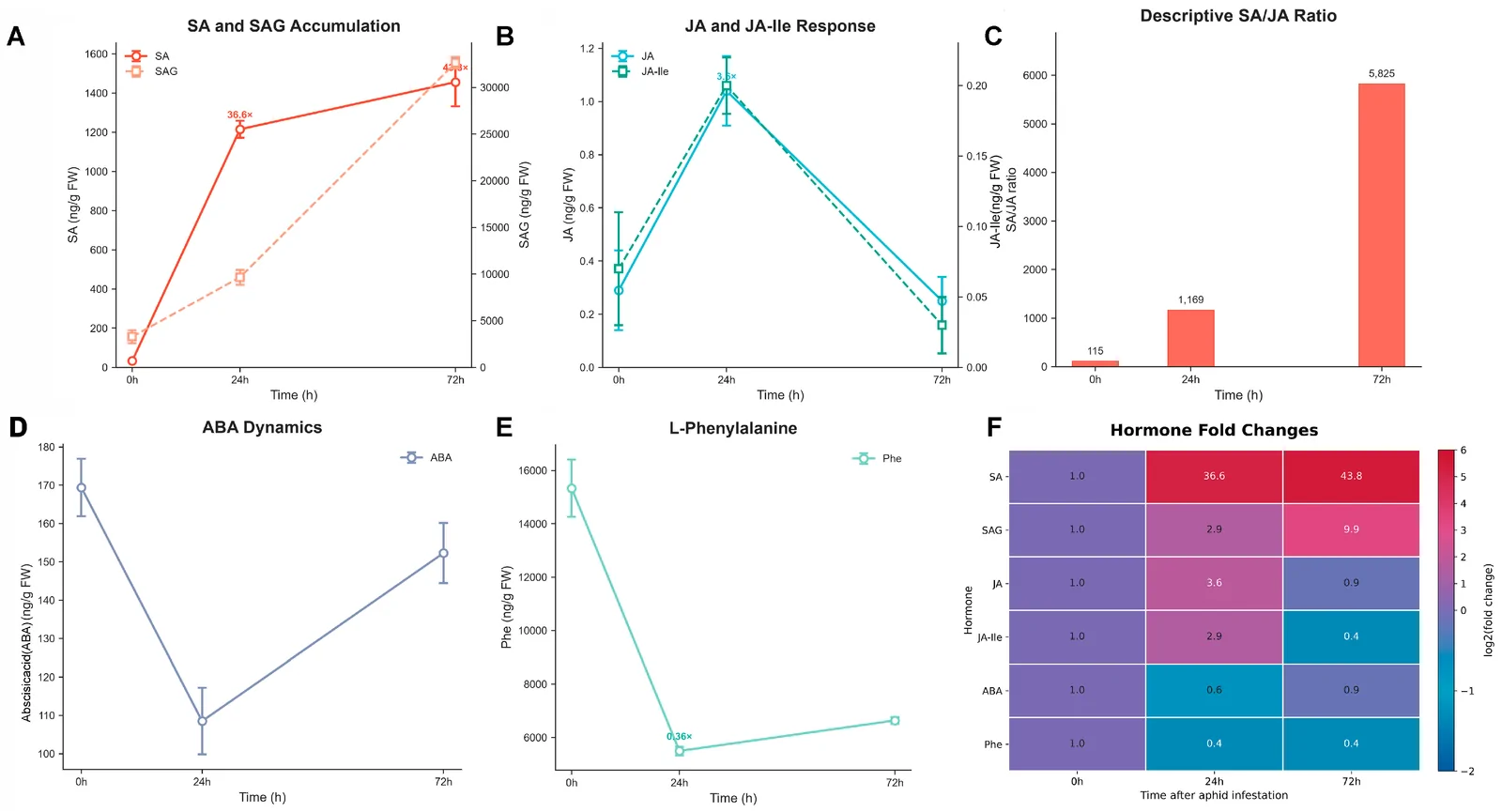
Phytohormone dynamics in *L. barbarum* under aphid stress. (**A**) Accumulation dynamics of SA and its glycosylated form SAG over the time course of aphid feeding. (**B**) JA and its bioactive derivative JA-Ile exhibited a transient peak at 24 h followed by a rapid decline. (**C**) Descriptive SA/JA ratio over the time course. The ratio was calculated from mean hormone concentrations and was not statistically tested; it is presented as a descriptive index suggesting a possible trend toward SA-dominated signaling. (**D**) Abscisic acid (ABA) levels showed a “checkmark”-shaped fluctuation, with a dynamic trend roughly opposite to that of the JA/JA-Ile response. (**E**) L-phenylalanine abundance decreased at 24 h and then rebounded, showing a dynamic trend roughly opposite to that of the JA/JA-Ile response. (**F**) Heatmap of hormone fold changes, with colors encoding log_2_ (fold change) and cell numbers reporting untransformed fold changes. Error bars in panels (**A**–**D**) represent SD (*n* = 3); ratios and fold changes were calculated from mean hormone concentrations.

Given that *NLR*-associated signaling often intersects with phytohormone pathways, and that transcriptomic analysis showed that *L. barbarum* mounted a more focused defense-related program, we further focused on this resistant accession and profiled the temporal dynamics of key defense hormones to resolve the signaling events underlying the transcriptional and *NLR* responses. To this end, we performed targeted phytohormone profiling at 0, 24, and 72 h; compared with the time points used for transcriptome analysis, this design included an additional early time point at 24 h to capture transient signaling events. Among the defense-related hormones examined, SA showed the most pronounced response. Free SA rose from 33.24 ng/g at 0 h to 1215.40 ng/g at 24 h (36.6-fold) and continued to increase to 1456.17 ng/g at 72 h (43.8-fold) (Figure 6A; Table S9). SAG accumulated in parallel (9.9-fold at 72 h), indicating active SA homeostasis through glycosylation (Figure 6A) [50].

By contrast, JA and JA-Ile exhibited a transient burst (Figure 6B), whereas SA continued to accumulate (Figure 6A). Descriptively, the SA/JA ratio increased over the time course (Figure 6C). The sustained accumulation of SA and the increase in the SA/JA ratio were opposite to the transient JA/JA-Ile burst. We hypothesize that these opposing trends may reflect a potential metabolic reconfiguration during early defense, possibly involving coordination between hormone signaling and the availability of precursors for defense-related secondary metabolism.

It is worth noting that ABA levels showed a “checkmark”-shaped fluctuation, decreasing at 24 h and then rebounding (Figure 6D); L-phenylalanine abundance similarly declined at 24 h and gradually recovered thereafter (Figure 6E), with both dynamic trends roughly opposite to the transient JA/JA-Ile burst, suggesting a potential metabolic reconfiguration during early defense that may reflect coordination between hormone signaling and precursor availability for defense-related secondary metabolism. Corresponding hormone fold changes are summarized in Figure 6F. To explore the relationship between hormone dynamics and defense gene responses, we calculated a directional hormone–gene response index (Figure S5A). Using this index, SA showed positive directional values with *PR4*, *LOX*, *GST*, and *WRKY70*-like genes, and negative values with *CHS* and *PAL*. The corresponding hormone concentrations and gene-expression changes are shown in Figure S5B–D.

### 2.5. Coordinated miRNA Expression Dynamics Suggest Post-Transcriptional Regulation

**Figure 7:**
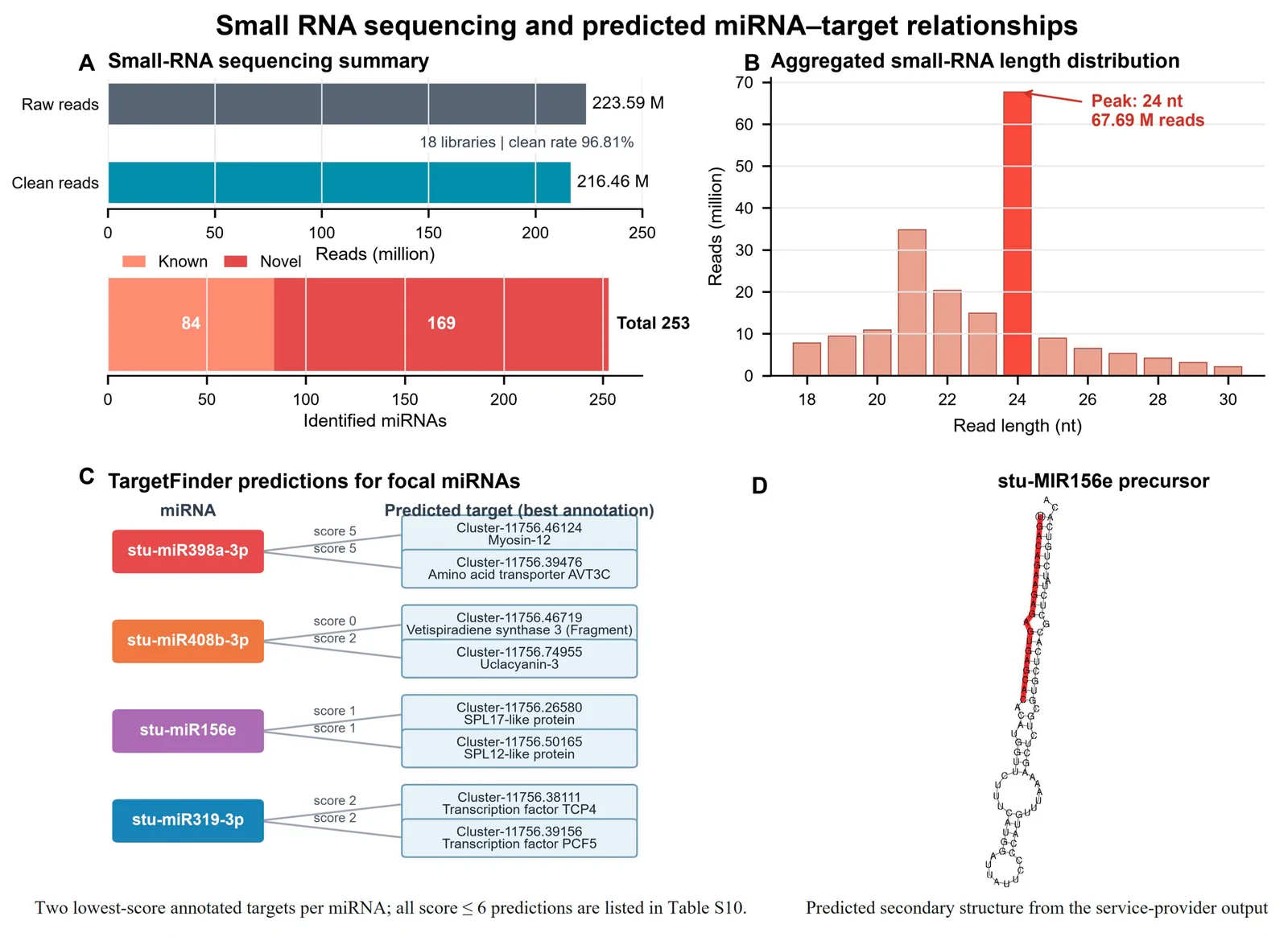
Small-RNA sequencing and predicted miRNA–target relationships. (**A**) Aggregated sequencing summary for 18 libraries, showing raw reads, clean reads, clean rate, and known, novel and total miRNA counts. (**B**) Aggregated 18–30-nt small-RNA length distribution across the 18 libraries; the peak was 24 nt (67,686,245 reads). (**C**) Computationally predicted target for *stu-miR398a-3p*, *stu-miR408b-3p*, *stu-miR156e*, and *stu-miR319-3p* using TargetFinder at a score cutoff of ≤6. All miRNA-target pairs shown are computational predictions only and have not been experimentally validated. For each miRNA, the panel displays at most two lowest-score targets with non-empty annotations; ties were ordered by annotation E-value and target ID. (**D**) Predicted secondary structure of the identified *stu-miR156e* precursor. These computationally predicted miRNA-target pairs represent candidates for downstream experimental validation.

To explore the potential role of miRNAs in post-transcriptional regulation during aphid defense, we analyzed 18 small-RNA libraries from *L. barbarum* collected at 0, 8, 24, 36, 48, and 72 h post-infestation (three biological replicates per time point). A total of 223,589,882 raw reads and 216,462,280 clean reads were obtained (96.81% clean rate), and 253 miRNAs were identified, including 84 known and 169 novel miRNAs (Figure 7A). The aggregated 18–30-nt length distribution peaked at 24 nt (67,686,245 reads; Figure 7B). Among the differentially expressed miRNAs (|log_2_FC| ≥ 1, FDR < 0.05), two showed peak abundance at 24 h coinciding with the JA burst: *stu-miR398a-3p* increased from 2224 TPM at 0 h to 23,673 TPM at 24 h (10.64-fold), and *stu-miR408b-3p* increased approximately 2.01-fold. By contrast, *stu-miR156e* reached its highest mean abundance at 36 h (2.09-fold relative to 0 h), whereas *stu-miR319-3p* increased 1.60-fold at 24 h but declined below baseline by 72 h. To identify potential targets of these miRNAs, we performed TargetFinder predictions with scores ≤6, which predicted candidate transcript targets for the four focal miRNAs (Figure 7C; Table S10). The predicted secondary structure of the *stu-miR156e* precursor is shown in Figure 7D. Although these miRNA-target pairs have not been experimentally confirmed, these computationally predicted interactions suggest candidate regulatory relationships for downstream experimental validation.

## 3. Discussion

This study presents an integrated multi-omics framework for the *Lycium*-aphid system by combining comparative transcriptomics, time-series phytohormone profiling in the resistant accession, small RNA sequencing, and genome-wide *NLR* analysis within a unified biological model. To our knowledge, limited information is currently available on how phytohormones, miRNAs, and *NLR* responses are temporally coordinated in woody perennials under aphid attack, and few studies have directly compared closely related *Lycium* accessions with contrasting resistance phenotypes. Through this integrative approach, we sought to move beyond a descriptive inventory of differentially expressed genes toward a systems-level understanding of how aphid defense is orchestrated in *L. barbarum*—from early signaling events to transcriptional reprogramming and post-transcriptional modulation. The hormone and small-RNA time-course analyses were performed in the resistant accession *L. barbarum*; the temporal patterns described here therefore reflect the response of this accession rather than a direct genotype-wise comparison between the two accessions.

A key observation of our study was that the resistant accession *L. barbarum* activated fewer DEGs (3441) than the susceptible *L. chinense* (8397), yet these genes were more strongly enriched in defense-related pathways. This pattern is consistent with the concept that effective defense may depend on the precision and focus of pathway activation rather than the sheer number of responsive genes, as documented in the sorghum–sugarcane aphid (SCA; *Melanaphis sacchari*) system [51]. In *L. barbarum*, *CHS* and *PAL* were down-regulated, whereas several *WRKY70-*, *PR-*, *LOX-*, *GST*-, and *PTI5*-related candidates were induced, indicating selective rather than uniform pathway activation. Because only one genotype per species was assessed, these differences may also reflect accession background effects. Moreover, DEG numbers can be substantially influenced by technical and analytical factors, including cross-species mapping bias, sequencing depth, genome annotation quality, normalization procedures, and statistical threshold selection. Therefore, the “less-but-more-focused” pattern is better regarded as a hypothesis-generating observation suggesting a possible resource-efficient defense mechanism in perennials, rather than definitive evidence for a distinct defense strategy. Stronger mechanistic conclusions will require comparable experimental designs and functional validation of key defense components. To gain deeper insight into the co-expression network architecture underlying this selective reprogramming, we applied WGCNA and identified the turquoise module as the co-expression module most tightly associated with stress treatment (r = 0.981). This module contained receptor-like kinases (RLKs), MAPK signaling components, and phenylpropanoid biosynthesis genes—consistent with a possible association with defense-related processes. Our network analysis further revealed that the turquoise module’s hub genes include a *CRK25*-like *RLK* candidate (*LOC132607248*) and multiple *WRKY*-related transcription factors, suggesting that *RLK*-mediated extracellular signal perception and *WRKY*-driven transcriptional reprogramming are tightly coordinated in the resistant accession. The prominence of *RLKs* in this module is noteworthy: these transmembrane receptors function as early sensors of extracellular signals [52,53], and together with intracellular NLR receptors, they constitute the two-tiered plant immune perception system [45,46]. Our data suggest that this coordinated *RLK-WRKY* module may be a key feature of the resistance mechanism in *L. barbarum*, a hypothesis that can now be tested through targeted gene silencing or overexpression experiments.

SA is a central regulator of plant immunity and systemic acquired resistance [54]. Our time-series phytohormone profiling further showed that, within this study’s time course, SA was also the most responsive hormone: its accumulation reached 43.8-fold at 72 h, making it the most prominent hormone-associated change across the time course. Concurrently, JA and JA-Ile exhibited a transient burst at 24 h (3.6- and 2.8-fold over baseline, respectively), followed by a rapid return to near baseline levels. Our data provide a detailed kinetic description of this SA-JA antagonistic trajectory in goji, showing that the SA/JA ratio increases continuously over 72 h. This temporal offset—SA rising and persisting, JA peaking and then rapidly declining—is consistent with the classical SA-JA antagonistic framework [25,55,56] and suggests a possible trend toward SA-dominated signaling in *L. barbarum*.

In addition to these hormonal dynamics, we uncovered an unexpected metabolic signature associated with the early defense response. The opposite temporal trajectories of ABA and L-phenylalanine relative to the JA/JA-Ile burst raise the possibility of a coordinated metabolic reconfiguration during early defense. We hypothesize that the transient decline in ABA at 24 h may relieve its antagonistic effect on JA signaling [56], thereby facilitating the rapid activation of JA-dependent defenses—a pattern consistent with the established role of ABA as a negative modulator of JA-regulated responses [56]. The depletion of L-phenylalanine may also reflect its consumption for the synthesis of phenylpropanoid-derived defense metabolites, consistent with reports that phenylpropanoid pathways are activated and metabolic flux is redirected during aphid infestation [54]. The return of both metabolites toward baseline at 72 h, together with the sustained SA accumulation and the decline of *CHS* and *PAL* transcripts, suggests a transition from an early JA-driven rapid defense phase to a sustained SA-dominated signaling, accompanied by a rebalancing of primary and secondary metabolism [56,57]. This metabolic reconfiguration hypothesis, while requiring further testing, provides a potential framework for understanding the metabolic costs of defense in perennial plants.

L-Phenylalanine and phenylpropanoid-associated transcripts also changed during the time course. L-Phenylalanine declined to 0.36-fold of baseline at 24 h, a change consistent with, but not demonstrating, altered phenylpropanoid metabolism, but this does not establish a causal relationship. Although SA biosynthesis can proceed via the phenylalanine route [58,59], the observed downregulation of *CHS* and *PAL* is more consistent with selective branch modulation than with global transcriptional activation of the entire phenylpropanoid pathway, suggesting a reconfiguration of metabolic flux. This pattern aligns with the general strategy by which plants coordinate hormonal signaling with secondary metabolism during defense [24,60,61], and with growth–defense trade-off frameworks [62].

Beyond phytohormonal regulation, our small-RNA time-series revealed a set of miRNAs whose expression dynamics are tightly coupled with the hormonal shift. The six-time-point small-RNA series revealed that *stu-miR398a-3p* reached its highest abundance at 24 h, coinciding temporally with the JA peak and early SA accumulation [63]. The observed temporal coupling between *miR398a-3p*/*miR319-3p* and the SA/JA shift, together with the known functions of *miR398* in regulating ROS homeostasis and JA-related defense genes [63] and *miR319* in modulating JA synthesis via TCP targets [63], raises the hypothesis of a possible regulatory circuit in which these miRNAs may act downstream of or in parallel with hormonal signals. *Stu-miR156e* peaked at 36 h rather than 72 h and Target Finder predicted *SPL17*-like and *SPL12*-like transcripts among its lowest-scoring annotated targets, consistent with the established *miR156–SPL* regulatory axis [29]. *Stu-miR319-3p* increased at 24 h and declined below baseline at 72 h; *TCP4* and *PCF5* were among its lowest-scoring annotated predicted targets, consistent with reported *miR319–TCP* links to jasmonate regulation [24]. Although these miRNA-target relationships remain computational predictions [64,65] requiring further validation by degradome sequencing, 5′ RACE, or reporter assays, the temporal coupling of miRNA peaks with the JA burst and early SA accumulation suggests that miRNA-mediated post-transcriptional regulation may act in concert with hormone signaling to fine-tune defense outputs in goji. This regulatory layer has received limited attention in woody perennials and warrants further investigation. Our genome-wide HMM-based analysis identified 151 candidate *NLR* genes in the *L. barbarum* reference genome, of which 18 were up-regulated in *L. barbarum* under the stated threshold but did not meet the same threshold in *L. chinense*. These candidates include genes encoding *TIR-NBS-LRR* and *CC-NBS-LRR* architectures, which fall within the major classes of plant NLR immune receptors [45,46]. Notably, the 18 *L. barbarum*-biased aphid-responsive *NLRs* were preferentially up-regulated in the same accession that exhibited sustained SA accumulation and SA-dominated signaling. This correlation, while descriptive, raises the possibility that these *NLR* candidates may function in coordination with the SA pathway, either as downstream components or as modulators of SA signaling [66]—a hypothesis that can be tested through targeted perturbation experiments, as MPK3/MPK6 have been shown to regulate SA signaling by modulating *NLR* expression during plant immunity [66].

**Figure 8:**
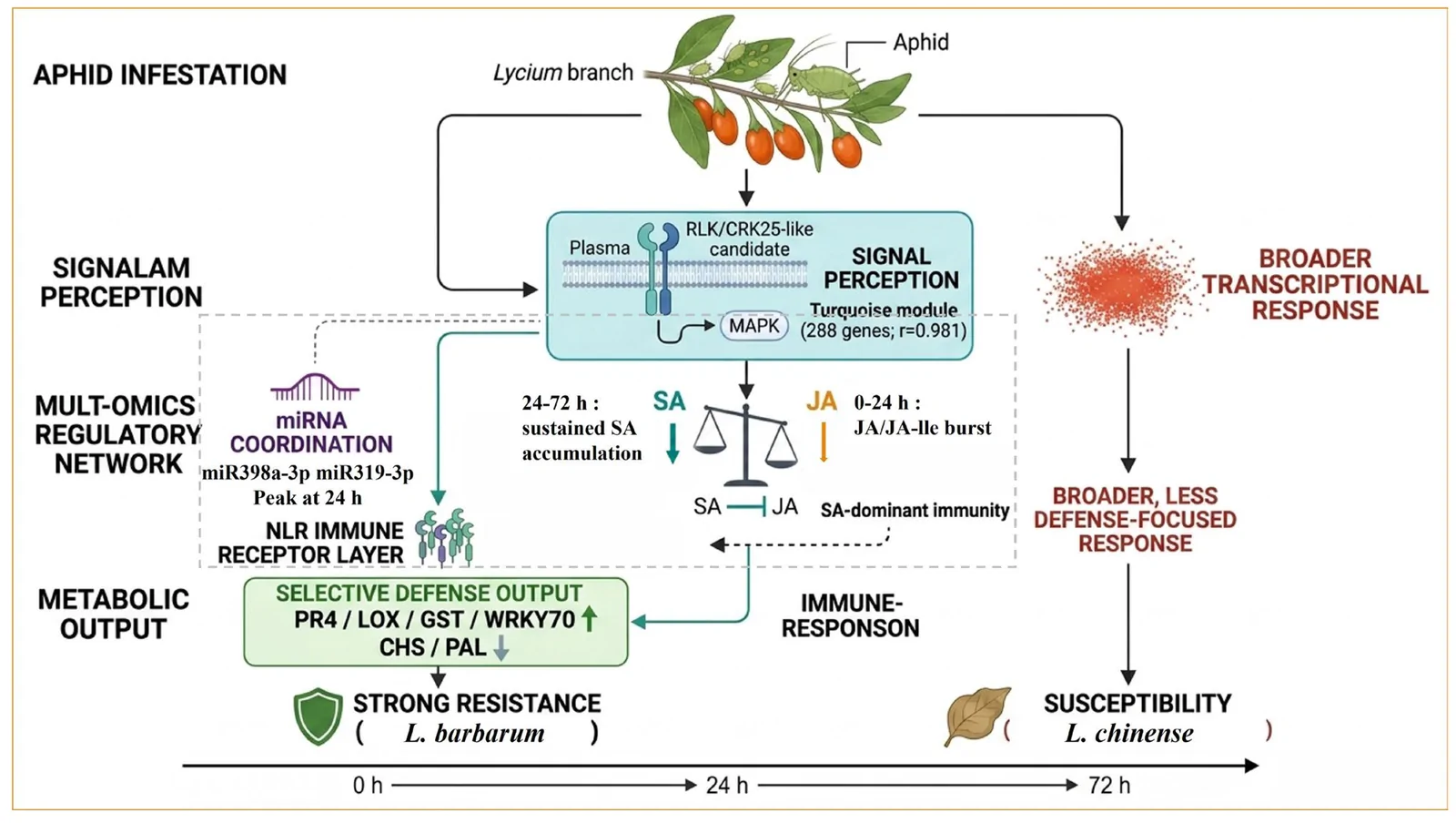
An integrated working model of aphid defense regulation in goji. Aphid feeding is initially perceived by *RLK/CRK25* and MAPK components (turquoise module), initiating intracellular hormone signaling. In the resistant accession *L. barbarum*, hormone and miRNA time-course data reveal a pronounced temporal shift in defense responses: a transient JA burst at 24 h, accompanied by decreased ABA and L-phenylalanine depletion, followed by sustained SA accumulation and an increased SA/JA ratio by 72 h, indicating a transition from JA-dominated to SA-dominated signaling. *miR398a-3p* and *miR319a-3p* peak at 24 h, coinciding with the initiation of the JA-to-SA transition and suggesting their possible involvement in coordinated hormonal regulation. Defense outputs include induction of *PR4/LOX/GST/WRKY70* and downregulation of *CHS/PAL*, reflecting a focused transcriptional strategy. At the *NLR* level, 18 *L. barbarum*-biased candidates are preferentially upregulated under SA-dominated signaling, implying a functional link to SA signaling. Together, transcriptomic, co-expression module, and *NLR* analyses indicate that resistant *L. barbarum* exhibits a “less-but-more-focused” defense pattern, whereas susceptible *L. chinense* mounts a broader but less targeted response. Solid arrows denote relationships directly supported by experimental evidence in this study; dashed arrows denote hypothesized or inferred relationships, including potential miRNA-hormone interactions, *NLR*-SA crosstalk, and other regulatory associations not experimentally validated. Note that the hormone and miRNA time courses were performed only in the resistant accession *L. barbarum*; therefore, the corresponding temporal trends in the figure represent this accession alone and should not be directly interpreted as genotypic differences in temporal dynamics between the two accessions. Comparisons between the two accessions are based mainly on transcriptomic, co-expression module, and *NLR* data.

Integrating these multi-layer data, we propose an association-supported working model (Figure 8) in which a transient JA response, sustained SA accumulation, a stress-associated turquoise module, miRNA-associated regulation, and accession-biased *NLR* responses define candidate layers of aphid defense in *L. barbarum*. A notable feature of this model is the temporal integration of these layers—showing how early transient signals (JA, miRNAs at 24 h) give way to sustained responses (SA, NLRs at 72 h)—and the identification of *L. barbarum*-biased components at both the transcriptional and receptor levels. Functional validation is required to establish causality.

Several limitations should be acknowledged. First, the miRNA–target interactions reported here are computational predictions and require validation through degradome sequencing, 5′ RACE, or reporter assays. Second, functional characterization of candidate hub genes—including the *CRK25*-like *RLK*, *WRKY*-related transcription factors, and *L. barbarum*-biased aphid-responsive *NLRs*—is also needed to establish their causal roles in aphid resistance. At the experimental design level, the hormone and small RNA time-course analyses were performed only in *L. barbarum*; although time-matched uninfested controls were included at each time point in this accession, comparable time-series data from *L. chinense* would strengthen accession-level inference and provide a more complete picture of how hormonal and post-transcriptional dynamics differ between the two accessions. The cross-accession transcriptomic comparison was further limited to a single time point (72 h), and earlier time points might capture additional regulatory events preceding the observed transcriptional program. Another limitation is that cross-species mapping to the *L. barbarum* reference genome may introduce bias, particularly for highly variable gene families such as *NLRs*; thus, accession-biased expression is interpreted conservatively as transcriptional bias rather than species-specific genomic differences. Finally, the hypotheses regarding ABA-mediated JA antagonism and metabolic reconfiguration are based on correlative patterns and require functional testing through hormone perturbation or gene knockout experiments. Despite these limitations, the integrated multi-omics approach provides a foundation for understanding aphid defense in *Lycium* and prioritizes candidate targets for future functional validation and marker-assisted breeding.

From a practical perspective, the candidate genes and regulatory modules identified here—particularly the 18 *L. barbarum*-biased *NLRs* and the *CRK25*-like *RLK*—provide potential targets for marker-assisted selection in goji breeding programs. Growers could ultimately benefit from aphid-resistant cultivars that reduce reliance on chemical insecticides. Future work should focus on functional validation of these candidates through transient expression or virus-induced gene silencing, and on extending the time-course analyses to *L. chinense* to enable direct comparative inference between resistant and susceptible genotypes.

## 4. Materials and Methods

### 4.1. Plant Materials, Insect Rearing

Plants of the susceptible wolfberry variety *Lycium chinense* Mill. and the *Lycium barbarum* ‘Ningqi-10’ were planted at the Tree Garden of the Inner Mongolia Academy of Forestry Sciences (40°48′ N, 111°42′ E, altitude 1056 m) and managed under conventional field conditions. For indoor experiments, plants were propagated from the same mother plant by cuttings to ensure genetic homogeneity.

The goji-specialized strain of *Aphis gossypii* (hereinafter referred to as *A. gossypii*) was collected from goji-growing areas in Inner Mongolia and reared on *L. chinense* without exposure to any insecticides. A monoclonal lineage of each species was established through the isolation and single-rearing of a single aphid, and the resulting colonies were propagated on *L. chinense*. All plants and aphids were maintained in a climate chamber at 22 ± 2 °C, 60–70% relative humidity, a photoperiod of 10 h light/14 h dark, and a light intensity of 2000 Lux.

### 4.2. Aphid Bioassays

Apterous adult aphids from a single clonal lineage were transferred onto susceptible goji plants and first-instar nymphs produced within 24 h were collected for subsequent experiments. These nymphs were then individually reared in clip cages on *L. chinense* and *L. barbarum* ‘Ningqi-10’ plants. Molting, fecundity, and other life-history traits were recorded daily until adult mortality. Each treatment consisted of three biological replicates, with 25 aphids per replicate. Survival data were analyzed using the Kaplan–Meier method, and survival curves were compared by the log-rank (Mantel–Cox) test.

Life cycle parameters were calculated as follows: the net reproductive rate, $R_{0}=\sum l_{x}m_{x}$, the mean generation time, $T=\sum xl_{x}m_{x}/R_{0}$, the intrinsic rate of increase, $r_{m}=(\ln R_{0})/T$, and the finite rate of increase, $\lambda =e^{r_{m}}$ and population doubling time $t=ln2/r_{m}$. In the equations, x is the age in days, $l_{x}$ is the survival rate at age x, and $m_{x}$ is the number of newborn nymphs produced per live adult at age x [67].

### 4.3. Experimental Design

The goji-specialized strain of *A. gossypii* was inoculated onto *L. chinense* and reared for at least 20 generations to establish an aphid population for subsequent experiments. Prior to bioassays, aphids were starved for 12 h.

Four transcriptomic treatment groups were designed, each with three biological replicates: uninfested *L. barbarum* (control, LbCK), aphid-infested *L. barbarum* (LbST), uninfested *L. chinense* (control, LyCK), and aphid-infested *L. chinense* (LyST). For infested plant, 30 apterous adults were transferred onto fully expanded leaves, while control plants received no aphids. All plants were maintained under identical conditions, and leaf tissues were collected from both infested and uninfested plants at 72 h after the start of the experiment. All visible aphids were carefully removed from the leaf surface prior to sampling.

To further resolve the temporal dynamics of defense signaling, targeted phytohormone profiling and small RNA sequencing were performed exclusively on *L. barbarum*. For these time-course analyses, both aphid-infested and time-matched uninfested control plants were sampled at each time point: 0, 24, and 72 h for phytohormone profiling, and 0, 8, 24, 36, 48, and 72 h for small RNA sequencing. All samples were flash-frozen in liquid nitrogen and stored at −80 °C until further processing.

### 4.4. RNA Extraction and Quality Assessment

Total RNA was extracted from approximately 100 mg of frozen leaf tissue using TRIzol reagent (Invitrogen, Carlsbad, CA, USA) according to the manufacturer’s protocol. RNA quality and concentration were assessed by 1% agarose gel electrophoresis, spectrophotometry (NanoDrop 2000; Thermo Fisher Scientific, Waltham, MA, USA), and microfluidic electrophoresis (Agilent 2100 Bioanalyzer; Agilent Technologies, Santa Clara, CA, USA). Only samples with an A260/A280 ratio between 1.8 and 2.1, an A260/A230 ratio greater than 2.0, and an RNA integrity number (RIN) ≥ 7.0 were used for subsequent transcriptome and small RNA library construction.

### 4.5. Transcriptome Sequencing and Data Processing

Transcriptome libraries were constructed using poly(A)-selected RNA and sequenced on an Illumina NovaSeq 6000 platform in paired-end 150-bp mode. Raw reads were filtered using fastp (v1.3.0) [68]. For consistent cross-accession quantification within a common annotation coordinate system, reads from both accessions were aligned to the *L. barbarum* reference assembly Lba_v1.0 using HISAT2 (v2.2.2) [69]. Only uniquely mapped reads with MAPQ ≥ 30 were retained. This shared-reference strategy enables comparison between the two accessions within a common annotation coordinate system but may introduce cross-accession mapping bias, particularly for highly variable gene families such as *NLRs*. In this study, “*L. barbarum*-biased” refers to expression bias under the current cross-species mapping strategy. Gene-level counts were analyzed with DESeq2 (v1.44.0) [70]. Within-accession aphid responses were evaluated using the LbST versus LbCK and LyST versus LyCK contrasts, with differentially expressed genes defined as |log_2_FC| ≥ 1 and FDR < 0.05. To formally test whether aphid-induced responses differed between accessions, we fitted a factorial model including genotype, treatment, and their interaction (~genotype + treatment + genotype: treatment). The interaction coefficient represented the contrast (LyST − LyCK) − (LbST − LbCK). Genes with an interaction FDR < 0.05 were considered to show statistically supported genotype-dependent responses; an additional |interaction log_2_FC| ≥ 1 threshold was applied for the interaction-defined gene set used in downstream over-representation analysis. The terms “*L. barbarum*-biased” and “*L. chinense*-biased” are used descriptively for genes detected in only one of the two within-accession contrasts.

### 4.6. Phytohormone Quantification

Phytohormones were extracted and quantified by Wuhan MetWare Biotechnology Co., Ltd., Wuhan, China. Briefly, 50 mg of frozen leaf tissue was extracted with 1 mL of methanol/water/formic acid (15:4:1, *v*/*v*/*v*) containing 10 µL of a 100 ng mL^−1^ internal-standard mixture. Samples were vortexed for 10 min and centrifuged at 12,000 rpm for 5 min at 4 °C. The supernatant was concentrated, reconstituted in 100 µL of 80% methanol, and passed through a 0.22-µm filter. Phytohormones were separated on an ExionLC AD system equipped with an HSS T3 C18 column and quantified using a QTRAP 6500+ mass spectrometer in scheduled multiple-reaction-monitoring (MRM) mode, following established LC–MS/MS plant hormone quantification workflows [71,72].

### 4.7. WGCNA Co-Expression Network Construction

Genes with a coefficient of variation > 0.1 across all samples were retained for co-expression network construction using the WGCNA R package (v1.74) [44]. A signed hybrid network was constructed with a soft-thresholding power of 6 (scale-free R^2^ = 0.933). Modules were identified by hierarchical clustering based on topological overlap matrix (TOM)-based dissimilarity, with minModuleSize = 50 and mergeCutHeight = 0.25. Module eigengenes correlated with binary trait variables, including stress treatment, genotype, and their interaction, using Pearson correlation. Hub genes were ranked by intramodular connectivity (kWithin). GO biological-process and KEGG pathway annotations for the WGCNA hub-gene sets were summarized using the clusterProfiler framework (v4.6.2) [73]. Formal over-representation analyses were then performed separately for each WGCNA module and annotation database using a right-tailed hypergeometric test. The background consisted of WGCNA network genes with valid annotations for the corresponding database. *p* values were adjusted using the Benjamini–Hochberg procedure across all valid tested terms within each module–database combination, and an FDR < 0.05 was considered statistically significant. Given that only 12 RNA-seq samples were available in this study, the module–trait associations should be interpreted with caution, and the turquoise module should be regarded as a candidate stress-associated module rather than a definitively validated one.

### 4.8. Small RNA Sequencing and miRNA Identification

Small RNA libraries were constructed from *L. barbarum* samples collected at 0, 8, 24, 36, 48, and 72 h post-infestation, with three biological replicates per time point (18 libraries), and sequenced in single-end 50-bp mode. Known miRNAs were identified by alignment to miRBase v22.1 [64]. The “stu-” prefix in the miRNA names follows the miRBase naming convention based on sequence homology with *Solanum tuberosum* miRNAs deposited in miRBase. Novel miRNAs were predicted using miREvo (v1.0) [74] and miRDeep2 (v2.0.1.3) [75]. Candidate targets were predicted with TargetFinder (v1.7) [65] using a score cutoff of ≤6. Differential miRNAs expression was analyzed using DESeq2 (v1.44.0) with |log_2_FC| ≥ 1 and FDR < 0.05, applying the Benjamini–Hochberg correction for multiple testing. The raw small RNA-seq reads generated in this study have been deposited in the NCBI Sequence Read Archive (SRA) under BioProject accession PRJNA1504421.

### 4.9. NLR Gene Identification and Classification

Candidate *NLR* genes were systematically identified from the *L. barbarum* reference genome (Lba_v1.0) using an HMM-based domain search. Profile HMMs for the NB-ARC domain (PF00931), Toll/interleukin-1 receptor (TIR) domain (PF01584), leucine-rich repeat (LRR) domain (PF01535), and coiled-coil (CC) domain (PF18052) were downloaded from Pfam v35.0 [76]. The predicted proteome was scanned using hmmscan from HMMER3 v3.3.2 [77] with an E-value cutoff of 10^−5^. Genes containing significant NB-ARC domain hits were retained as candidate *NLR* genes. Based on the presence and combination of NB-ARC, TIR, CC, and LRR domains, the candidates were classified into four categories: TIR-NBS-LRR, CC-NBS-LRR/NB-ARC, NB-ARC-only, and NLR proteins with non-canonical domain combinations that do not match the major NLR classes (Table S7; Figure 5D). Table S7 provides the complete list of the 151 candidate *NLR* genes, including domain classes and DESeq2 statistics (log_2_FC and padj) for both *L. barbarum* and *L. chinense*.

### 4.10. Quantitative Real-Time PCR

Five defense-related genes were selected for RT-qPCR validation based on their differential expression patterns in the RNA-seq data, with *LbActin* as the internal reference gene. First-strand cDNA was synthesized from 1 µg of total RNA using the PrimeScript™ RT Reagent Kit with gDNA Eraser (TaKaRa, Kusatsu, Japan) according to the manufacturer’s protocol. QPCR was performed using TB Green™ Premix Ex Taq™ II (TaKaRa, Kusatsu, Japan) on a QuantStudio 5 system (Applied Biosystems, Waltham, MA, USA) under the following conditions: 95 °C for 30 s, followed by 40 cycles of 95 °C for 5 s and 60 °C for 30 s, with a final dissociation curve analysis to confirm amplification specificity. Relative expression levels were calculated using the 2^−ΔΔCt^ method [78]. Three independent biological replicates and three technical replicates per biological replicate were performed for each sample. Differences between control and aphid-stress treatments were analyzed using two-sided Welch’s *t*-test on log_2_-transformed relative-expression values, with Holm correction across the 10 planned comparisons. Primer sequences are listed in Table S6.

### 4.11. Statistical Analysis

All statistical analyses were performed in R v4.2.2 (R Foundation for Statistical Computing, Vienna, Austria). Hormone data were tested by one-way ANOVA with Tukey’s HSD post-hoc test after assessing normality (Shapiro–Wilk test) and homogeneity of variance (Levene’s test); when assumptions were violated, the Kruskal–Wallis test with Dunn’s correction was applied. Except for the RT-qPCR comparisons, which used Holm correction as described above, *p*-values from multiple-testing analyses were adjusted using the Benjamini–Hochberg method. For the exploratory matrix in Figure S5A, we calculated an unscaled directional hormone–gene response index as the product of the hormone log_2_ fold change between 72 h and 0 h and the corresponding DESeq2 gene log_2_ fold change at 72 h. Positive and negative values indicate changes in the same and opposite directions, respectively. This index was used for descriptive visualization. Figures were generated using ggplot2 v3.4.2 and pheatmap v1.0.12. The SA/JA ratio was calculated from mean hormone concentrations.

## 5. Conclusions

This study presents an integrated multi-omics dissection of aphid defense in *Lycium*, combining comparative transcriptomics, phytohormone profiling, small RNA sequencing, and genome-wide *NLR* analysis in a natural comparative system—*L. barbarum* (cv. Ningqi-10) versus susceptible *L. chinense*. The results define a temporally coordinated defense framework centered on phytohormone dynamics, with miRNAs and *NLRs* as cooperating regulatory layers.

Upon aphid attack, the resistant accession *L. barbarum* activated fewer DEGs (3441 vs. 8397) than *L. chinense*, yet these genes showed stronger enrichment in defense pathways, consistent with a more focused defense response. Given that only one genotype per species was compared, these differences may also reflect accession background effects and cannot be directly attributed to species specificity. Nevertheless, the “less-but-more-focused” pattern remains a valuable observation suggesting that perennials may adopt a resource-efficient defense strategy; however, because DEG numbers are sensitive to technical and analytical factors, this pattern is better regarded as a lead requiring further validation rather than definitive evidence. Phytohormone profiling in the resistant accession *L. barbarum* revealed a clear temporal split. As time-course data were available only for *L. barbarum*, these temporal patterns pertain to the resistant accession. JA and JA-Ile burst transiently at 24 h, while SA accumulated progressively to 72 h. Descriptively, the SA/JA ratio increased over the time course, suggesting a possible trend toward SA-dominated signaling. Concurrently, ABA declined and L-phenylalanine was depleted at 24 h—a pattern opposite to the JA burst and suggestive of early metabolic reconfiguration that may relieve JA antagonism and redirect phenylpropanoid precursors toward defense metabolites. The return of both metabolites to baseline at 72 h, together with SA persistence and downregulation of *CHS/PAL*, is consistent with a transition from an early JA-driven rapid defense phase to a sustained SA-dominated response in *L. barbarum*.

At the post-transcriptional level, *stu-miR398a-3p* and *stu-miR319-3p* peaked at 24 h, coinciding temporally with the JA burst and early SA accumulation. Their temporal coupling with the SA/JA shift, together with known functions of *miR398* in ROS/JA regulation and *miR319* in JA synthesis via TCP targets, suggests that these miRNAs may act downstream of or in parallel with hormonal signals. These associations provide candidate leads for understanding the regulatory role of miRNAs in goji defense, and their functional significance remains to be experimentally confirmed.

In the *NLR* layer, 151 candidate *NLR* genes were identified, of which 18 *L. barbarum*-biased *NLRs* were preferentially upregulated under SA-dominated signaling suggesting a possible functional link between SA signaling and *NLR*-mediated immunity in goji.

Collectively, these findings support a multi-layered model in which *RLK*/MAPK-mediated perception initiates a defense response that unfolds in two temporally distinct phases: an early phase at 24 h, characterized by a transient JA burst coupled with ABA/L-phenylalanine metabolic reconfiguration and miRNA regulation; and a sustained phase at 72 h, marked by progressive SA accumulation that drives SA-dominated signaling and coincides with the upregulation of *NLR* candidates. This framework integrates temporal hormone dynamics, miRNA-associated post-transcriptional regulation, and *NLR* immune receptor responses into a unified defense architecture. While several regulatory links remain hypotheses requiring functional validation, this study provides a molecular foundation for understanding aphid defense in *Lycium* and prioritizes candidate targets for resistance breeding.

In summary, this study defines a temporally coordinated defense framework in goji centered on SA/JA signaling, with miRNAs and *NLRs* as cooperating regulatory layers. For growers, these findings highlight the potential of breeding for enhanced SA-mediated defense and *NLR*-mediated immunity to reduce aphid damage. For future researchers, the candidate genes and regulatory modules identified here provide a foundation for functional validation and marker-assisted breeding in goji and related Solanaceae crops.

## Data Availability

The raw transcriptome (mRNA-seq) and small RNA-seq reads generated in this study have been deposited in the NCBI Sequence Read Archive (SRA) under BioProject accession PRJNA1504421. All other data supporting the findings of this study are available within the Article and its Supplementary Information.

## Supplementary Materials

The following supporting information can be downloaded at: https://www.mdpi.com/article/10.3390/plants15203097/s1, Table S1: Life table parameters of *A. gossypii* at *L.cheseines* and *L. barbarum*; Table S2: Complete differential-expression results for *L. barbarum* and *L. chinense* comparisons, including fold changes, FDR values, expression summaries, and available functional annotations; Table S3: WGCNA module–trait relationships and module sizes; Table S4: GO biological-process annotations and over-representation statistics for WGCNA hub-gene sets; Table S5: KEGG pathway annotations and over-representation statistics for WGCNA hub-gene sets; Table S6: RT-qPCR primer sequences and corrected candidate labels; Table S7: DESeq2 statistics(log_2_FC and padj); Table S8: Eighteen *L. barbarum*-biased aphid-responsive *NLR* candidates; Table S9: Phytohormone concentrations across time points in *L. barbarum*; Table S10: All TargetFinder-predicted targets (score ≤ 6) for *stu-miR398a-3p*, *stu-miR408b-3p*, *stu-miR156e*, and *stu-miR319-3p*. Figure S1. Transcriptomic differential expression and network analyses of two *Lycium* accessions at 72 h post-aphid infestation. (A) Numbers of up- and down-regulated DEGs in each accession. (B) Venn summary of shared and accession-biased DEGs between the two accessions. (C) Distributions of log_2_ fold changes for DEGs in each accession. DEGs were defined by |log_2_FC| ≥ 1 and FDR < 0.05. Figure S2. Defense-gene expression profiles in *L. barbarum* at 72 h after aphid infestation. Bars show mean FPKM values from three RNA-seq biological replicates for seven defense-related genes (*PR4*, *LOX*, *GST*, *CHS*, *PAL*, *WRKY70* and *PTI5*) in control and aphid-stressed samples. Fold-change labels above the paired bars summarize the magnitude and direction of the 72-h response. Figure S3. Parameter evaluation and module overview of WGCNA network construction. (A) Scale-free fit index (R^2^) and mean connectivity at different soft-thresholding powers. Power = 6 (R^2^ = 0.933) was selected for subsequent network construction. (B) The ten co-expression modules identified and their corresponding gene counts. Figure S4. RT-qPCR validation of selected defense-related genes. Bars represent the mean of three biological replicates, with error bars indicating SEM. Relative expression values are displayed on a log_2_ scale. Statistical analysis was performed using two-sided Welch’s *t*-tests on log_2_-transformed relative expression values, with Holm correction applied across the 10 planned comparisons. Asterisks indicate Holm-adjusted significance levels (* *p* < 0.05, ** *p* < 0.01, *** *p* < 0.001, **** *p* < 0.0001). Figure S5. Directional hormone–gene response index and related expression trends. (A) Directional hormone–gene response index. For each hormone-gene pair, the index is the signed product of the hormone log_2_ fold change (72 h vs. 0 h) and the corresponding DESeq2 gene log_2_ fold change at 72 h. Positive and negative values indicates changes in the same and opposite directions, respectively. This index is descriptive only and is not a formal correlation or statistical test. (B) Heatmap of hormone concentrations and selected defense gene expression over the time course. Colors indicate row z-scores, and cell labels show mean concentrations (ng g^−1^ fresh weight; *n* = 3 biological replicates). (C) DESeq2 log_2_ fold changes for selected defense-related genes at 72 h after aphid infestation relative to the control. *WRKY70*-like and *CRK25*-like correspond to *LOC132606951* and *LOC132607248*, respectively.
