# Supplementary material for: Multi-Omics Analysis Reveals Hormonal, miRNA-Associated, and NLR-Linked Aphid Defense in Lycium barbarum
Source: Plants (Basel). 2026 Oct 10;15(20):3097. doi: 10.3390/plants15203097 (PMC13708591; doi:10.3390/plants15203097)
Supplement: Supplementary file 1 [file plants-15-03097-s001.zip › Supplementary Figures.pdf]

FigS1

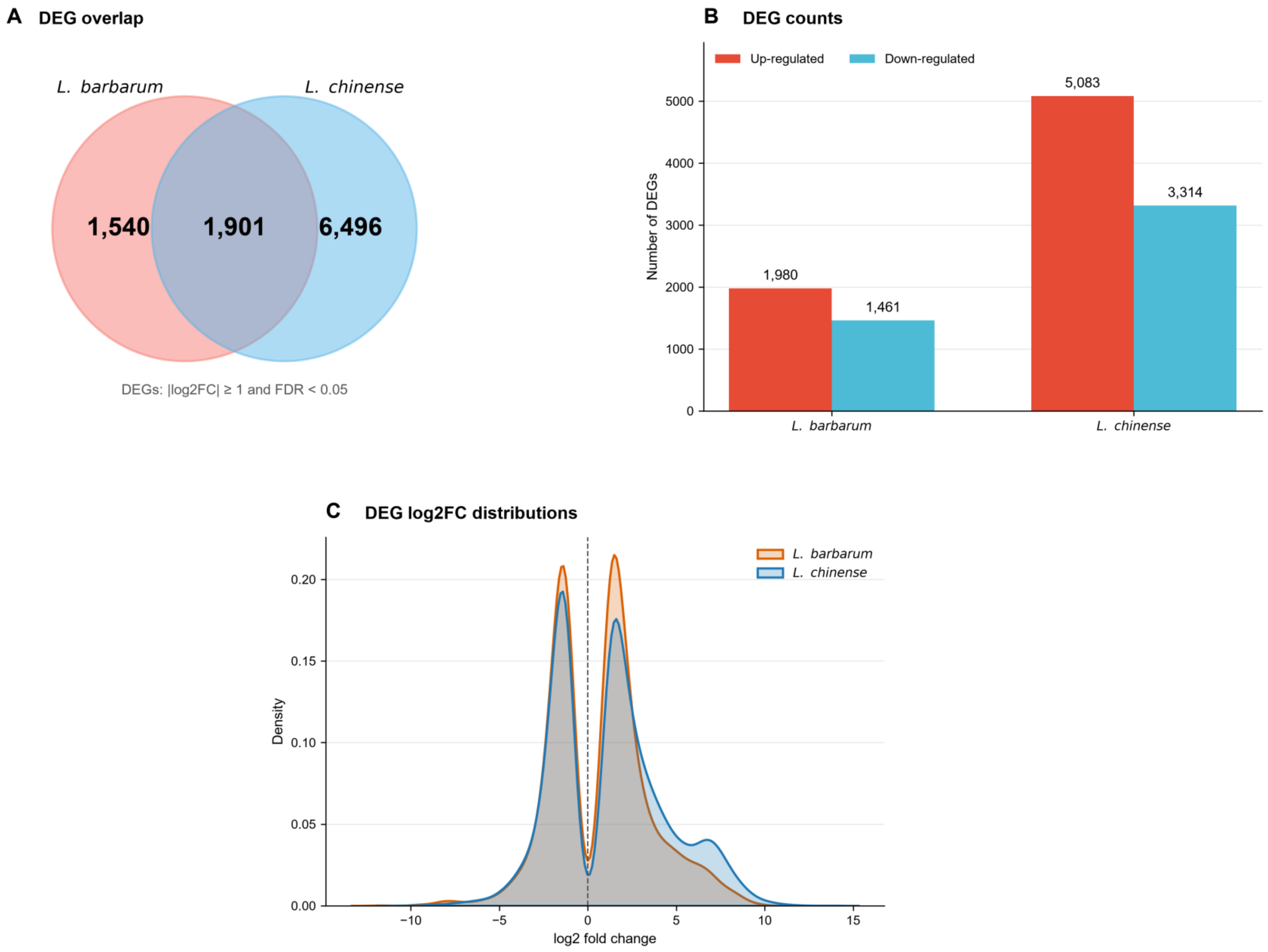
A DEG overlap
L. barbarum
L. chinense
1,540
1,901
6,496
DEGs: |log2FC| ≥ 1 and FDR < 0.05
B DEG counts
Up-regulated
Down-regulated
Number of DEGs
0
1000
2000
3000
4000
5000
1,980
1,461
5,083
3,314
L. barbarum
L. chinense
C DEG log2FC distributions
L. barbarum
L. chinense
Density
0.00
0.05
0.10
0.15
0.20
−10
−5
0
5
10
15
log2 fold change

Supplementary Figure S1. **Transcriptomic differential expression and network analyses of two Lycium accessions at 72 h post-aphid infestation.** (A) Numbers of up- and down-regulated DEGs in each accession. (B) Venn summary of shared and accession-biased DEGs between the two accessions. (C) Distributions of $\log_2$ fold changes for DEGs in each accession.DEGs were defined by $|\log_2 FC| \geq 1$ and FDR < 0.05.

FigS2

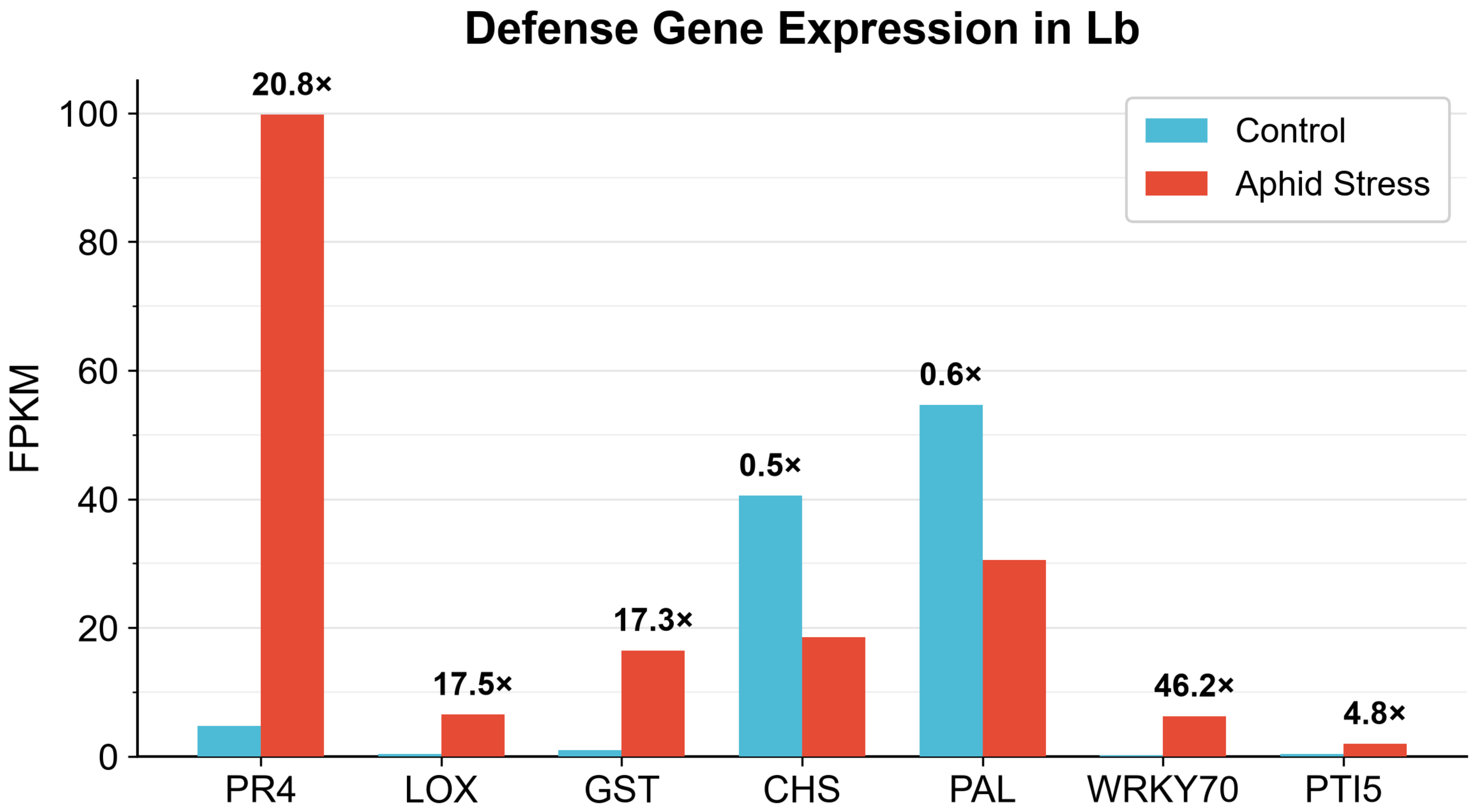

Defense Gene Expression in Lb
FPKM
100
80
60
40
20
0
20.8×
17.5×
17.3×
0.5×
0.6×
46.2×
4.8×
PR4
LOX
GST
CHS
PAL
WRKY70
PTI5
Control
Aphid Stress

Supplementary Figure S2. Defense-gene expression profiles in *L. barbarum* at 72 h after aphid infestation. Bars show mean FPKM values from three RNA-seq biological replicates for seven defense-related genes (*PR4*, *LOX*, *GST*, *CHS*, *PAL*, *WRKY70* and *PTI5*) in control and aphid-stressed samples. Fold-change labels above the paired bars summarize the magnitude and direction of the 72-h response.

FigS3

A

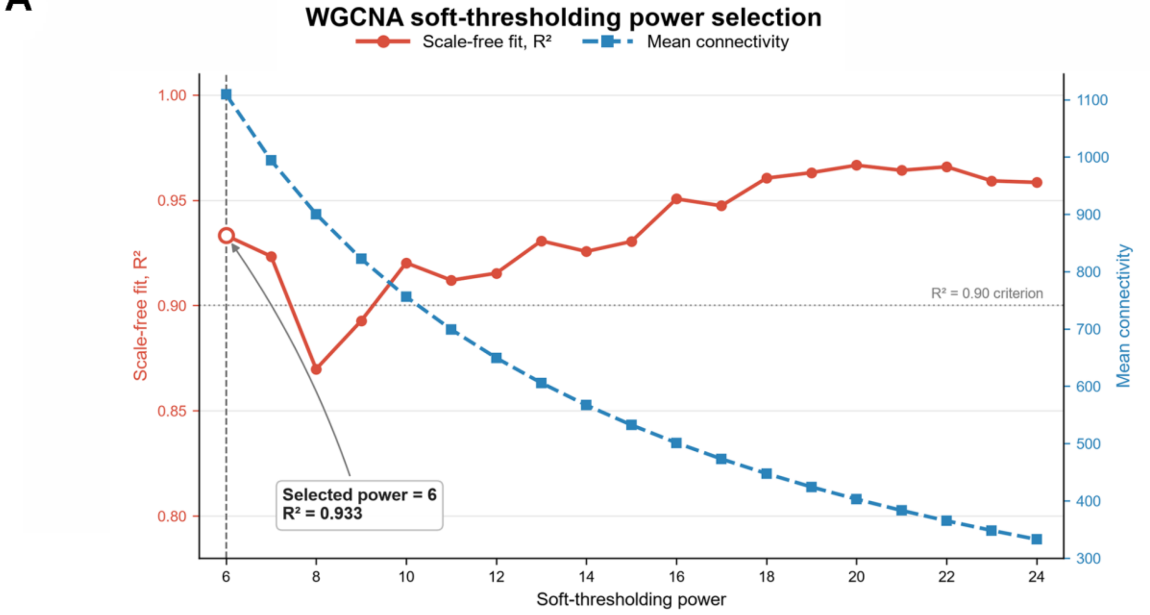

WGCNA soft-thresholding power selection
Scale-free fit, R²
Mean connectivity
Scale-free fit, R²
Mean connectivity
R² = 0.90 criterion
Selected power = 6
R² = 0.933
Soft-thresholding power
1.00
0.95
0.90
0.85
0.80
1100
1000
900
800
700
600
500
400
300
6
8
10
12
14
16
18
20
22
24

B

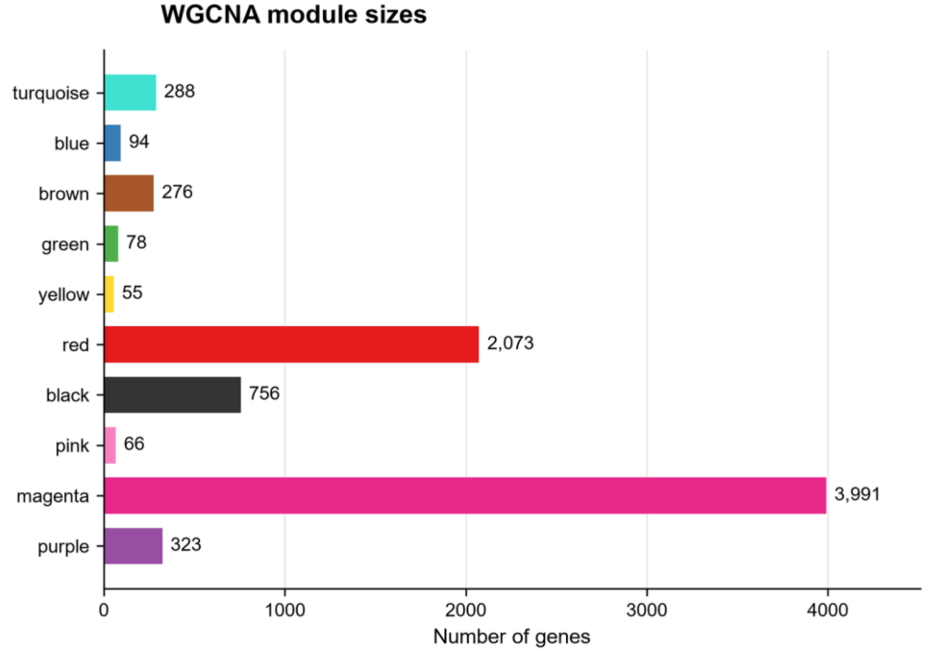

WGCNA module sizes
turquoise
288
blue
94
brown
276
green
78
yellow
55
red
2,073
black
756
pink
66
magenta
3,991
purple
323
0
1000
2000
3000
4000
Number of genes

Supplementary Figure S3. Parameter evaluation and module overview of WGCNA network construction.

(A) Scale-free fit index (R^2) and mean connectivity at different soft-thresholding powers. Power = 6 ($R^2 = 0.933$) was selected for subsequent network construction. (B) The ten co-expression modules identified and their corresponding gene counts.

FigS4

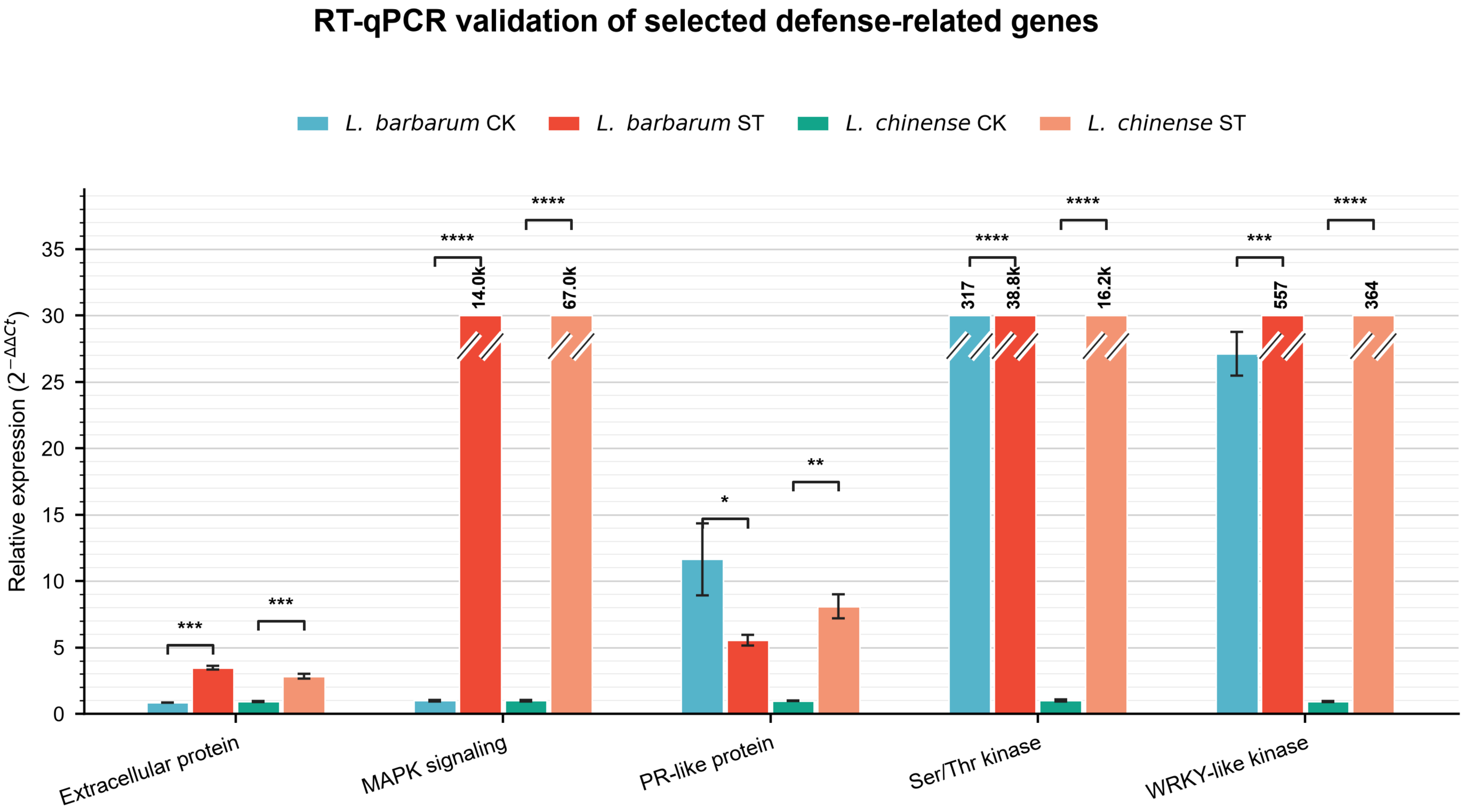

RT-qPCR validation of selected defense-related genes
L. barbarum CK
L. barbarum ST
L. chinense CK
L. chinense ST
Relative expression ($2^{-\Delta\Delta Ct}$)
0
5
10
15
20
25
30
35
***
***
****
****
14.0k
67.0k
*
**
****
****
317
38.8k
16.2k
***
****
557
364
Extracellular protein
MAPK signaling
PR-like protein
Ser/Thr kinase
WRKY-like kinase

Supplementary Figure S4. RT-qPCR validation of selected defense-related genes. Bars represent the mean of three biological replicates, with error bars indicating SEM. Relative expression values are displayed on a $\log_2$ scale. Statistical analysis was performed using two-sided Welch's t-tests on $\log_2$-transformed relative expression values, with Holm correction applied across the 10 planned comparisons. Asterisks indicate Holm-adjusted significance levels (*$p < 0.05$, **$p < 0.01$, ***$p < 0.001$, ****$p < 0.0001$).

FigS5

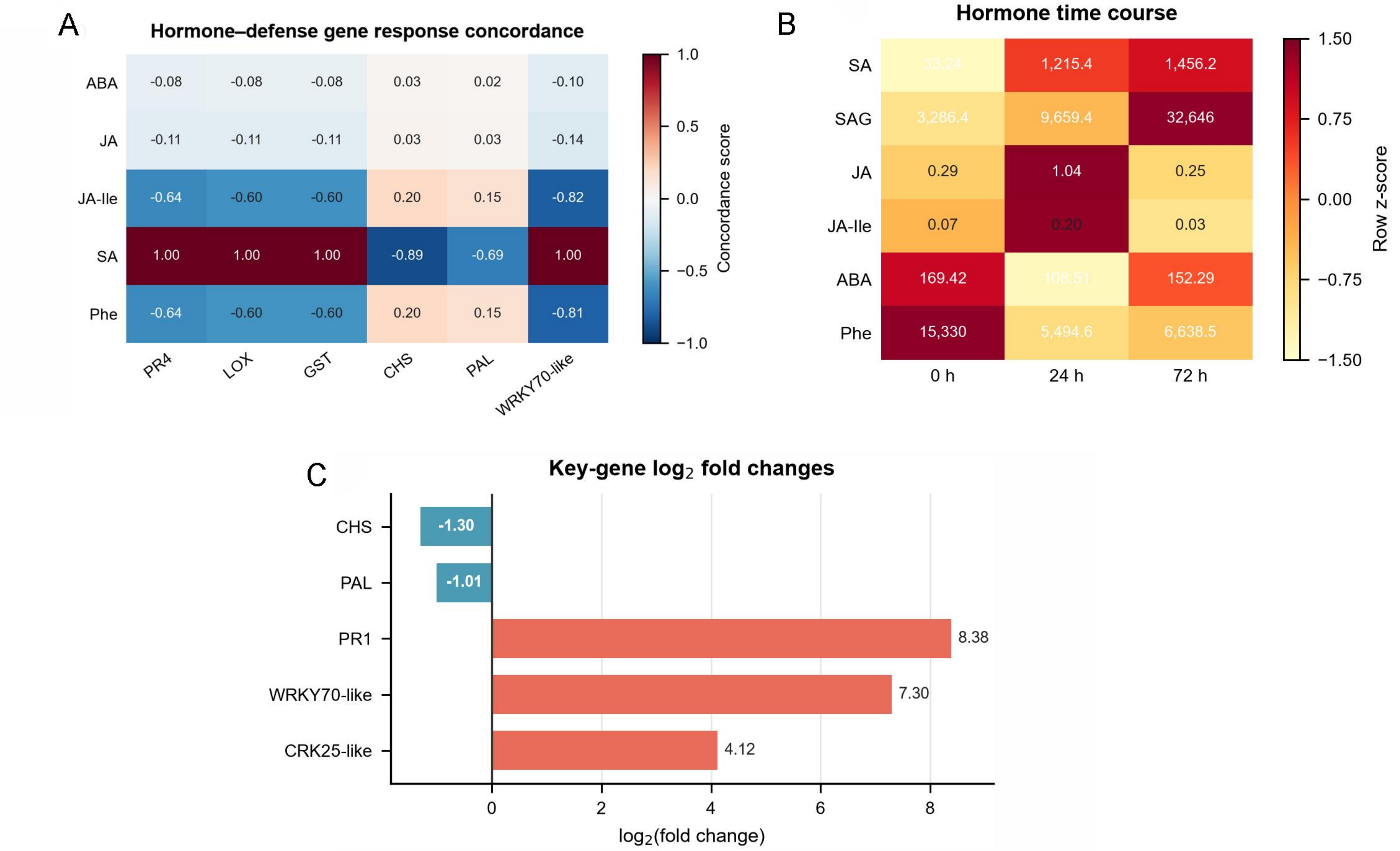
A
Hormone–defense gene response concordance
ABA
JA
JA-Ile
SA
Phe
PR4
LOX
GST
CHS
PAL
WRKY70-like
-0.08 -0.08 -0.08 0.03 0.02 -0.10
-0.11 -0.11 -0.11 0.03 0.03 -0.14
-0.64 -0.60 -0.60 0.20 0.15 -0.82
1.00 1.00 1.00 -0.89 -0.69 1.00
-0.64 -0.60 -0.60 0.20 0.15 -0.81
Concordance score
1.0
0.5
0.0
−0.5
−1.0
B
Hormone time course
SA
SAG
JA
JA-Ile
ABA
Phe
0 h
24 h
72 h
33.24 1,215.4 1,456.2
3,286.4 9,659.4 32,646
0.29 1.04 0.25
0.07 0.20 0.03
169.42 108.51 152.29
15,330 5,494.6 6,638.5
Row z-score
1.50
0.75
0.00
−0.75
−1.50
C
Key-gene log2 fold changes
CHS
PAL
PR1
WRKY70-like
CRK25-like
-1.30
-1.01
8.38
7.30
4.12
0
2
4
6
8
log2(fold change)

Supplementary Figure S5. Hormone dynamics and hormone-associated defense responses in *L. barbarum*. (A) Heatmap of co-trends between hormones and selected defense genes over the time course. Colors indicate row z-scores, and cell labels show mean concentrations (ng g^{-1} fresh weight; $n = 3$ biological replicates). (B) Directional response-concordance matrix quantifying the directional consistency between hormone changes and gene expression changes. Scores are calculated as the signed product of log_2(72 h/0 h hormone fold change) and the corresponding gene DESeq2 log_2 fold change, divided by 8 and bounded between −1 and +1; positive and negative values indicate concordant and opposing response directions, respectively. (C) DESeq2 log_2 fold changes for selected defense-related genes at 72 h after aphid infestation relative to the control. *WRKY70*-like and *CRK25*-like correspond to LOC132606951 and LOC132607248, respectively.
