# Supplementary material for: Multi-Omics Analysis Reveals Hormonal, miRNA-Associated, and NLR-Linked Aphid Defense in Lycium barbarum
Source: Plants (Basel). 2026 Oct 10;15(20):3097. doi: 10.3390/plants15203097 (PMC13708591; doi:10.3390/plants15203097)
Supplement: Supplementary file 1 [file plants-15-03097-s001.zip › Supplementary_Table S1_age-stage.pdf]

Supplementary Table S1. Life table parameters of *A. gossypii* at *L.cheseines* and *L. barbarum*.

Parameters	*L. chinense*	*L. barbarum*
r_0	42.60±2.58 a	13.53±0.82 b
T	12.87±0.02 a	11.72±0.10 b
r_m	0.29±0.01 a	0.21±0.02 b
λ	1.34±0.01 a	1.23±0.02 b
t	2.38 ±0.04 a	3.40±0.31 b

Data are presented as mean ± SEM (n = 3) with 25 individuals per replicate. Different lowercase letters within the same row indicate significant differences among treatments (one-way ANOVA with Tukey's HSD post-hoc test, $p < 0.05$), whereas the same letter denotes no significant difference.

Note: Net reproductive rate (R_o), intrinsic rate of increase (r_m), finite rate of increase (λ), mean generation time (T), and population doubling time (t).
